# Pneumococcal Vaccine for Adults Aged ≥19 Years: Recommendations of the Advisory Committee on Immunization Practices, United States, 2023

**DOI:** 10.15585/mmwr.rr7203a1

**Published:** 2023-09-08

**Authors:** Miwako Kobayashi, Tamara Pilishvili, Jennifer L. Farrar, Andrew J. Leidner, Ryan Gierke, Namrata Prasad, Pedro Moro, Doug Campos-Outcalt, Rebecca L. Morgan, Sarah S. Long, Katherine A. Poehling, Adam L. Cohen

**Affiliations:** ^1^National Center for Immunization and Respiratory Diseases, CDC, Atlanta, Georgia; ^2^National Center for Emerging and Zoonotic Infectious Diseases, CDC, Atlanta, Georgia; ^3^University of Arizona College of Medicine, Phoenix, Arizona; ^4^Department of Health Research Methods, Evidence and Impact, McMaster University, Hamilton, Ontario, Canada; ^5^Drexel University College of Medicine, Drexel University, Philadelphia, Pennsylvania; ^6^Wake Forest School of Medicine, Wake Forest University, Winston-Salem, North Carolina

## Abstract

**This report compiles and summarizes all published recommendations from CDC’s Advisory Committee on Immunization Practices (ACIP) for use of pneumococcal vaccines in adults aged ≥19 years in the United States. This report also includes updated and new clinical guidance for implementation from CDC:**

**Before 2021, ACIP recommended 23-valent pneumococcal polysaccharide vaccine (PPSV23) alone (up to 2 doses), or both a single dose of 13-valent pneumococcal conjugate vaccine (PCV13) in combination with 1–3 doses of PPSV23 in series (PCV13 followed by PPSV23), for use in U.S. adults depending on age and underlying risk for pneumococcal disease. In 2021, two new pneumococcal conjugate vaccines (PCVs), a 15-valent and a 20-valent PCV (PCV15 and PCV20), were licensed for use in U.S. adults aged ≥18 years by the Food and Drug Administration:**

**ACIP recommendations specify the use of either PCV20 alone or PCV15 in series with PPSV23 for all adults aged ≥65 years and for adults aged 19–64 years with certain underlying medical conditions or other risk factors who have not received a PCV or whose vaccination history is unknown. In addition, ACIP recommends use of either a single dose of PCV20 or ≥1 dose of PPSV23 for adults who have started their pneumococcal vaccine series with PCV13 but have not received all recommended PPSV23 doses. Shared clinical decision-making is recommended regarding use of a supplemental PCV20 dose for adults aged ≥65 years who have completed their recommended vaccine series with both PCV13 and PPSV23:**

**Updated and new clinical guidance for implementation from CDC includes the recommendation for use of PCV15 or PCV20 for adults who have received PPSV23 but have not received any PCV dose. The report also includes clinical guidance for adults who have received 7-valent PCV (PCV7) only and adults who are hematopoietic stem cell transplant recipients:**

## Introduction

Pneumococcal infections are caused by *Streptococcus pneumoniae* (pneumococcus), a gram-positive, facultative anaerobic bacterium. Pneumococcus can colonize the upper respiratory tract, most commonly in young children, and is transmitted to others through contact with respiratory droplets from a person with pneumococcal colonization in the upper respiratory tract (*1*). Certain persons with pneumococcal colonization might develop invasive pneumococcal disease (IPD) (*2*). IPD is infection of normally sterile sites, including pneumonia with bacteremia, meningitis, osteomyelitis, septic arthritis, and bacteremia without a focus of infection; examples of noninvasive disease include pneumonia without bacteremia, sinusitis, or otitis media. In adults, pneumococcal pneumonia is the most common type of pneumococcal disease, and pneumococcus is the most common bacterial cause of pneumonia that results in hospitalization (*3*).

Pneumococci are classified into serotypes depending on their capsular polysaccharide, which is a main virulence factor for pneumococcus (*4*). At least 100 pneumococcal serotypes were documented as of 2020 (*5*–*7*). During 2018–2019, approximately 60%–75% of all IPD in adults was caused by the 24 pneumococcal serotypes that were included in the formulations of commercially available polysaccharide conjugate vaccine (PCV) or pneumococcal polysaccharide vaccine (PPSV) vaccines (i.e., PCV13, PCV15, PCV20, and PPSV23) (Figure). Current pneumococcal vaccines use the pneumococcal capsular polysaccharides as antigens to generate serotype-specific antibodies, which facilitate serotype-specific clearance of pneumococci through opsonophagocytosis (*4*). PPSV23 (Pneumovax23) is a 23-valent vaccine that has been recommended for use since the 1980s for persons aged ≥2 years with certain underlying medical conditions and for adults aged ≥65 years (Table 1) (Figure). In 2000, the Advisory Committee on Immunization Practices (ACIP) recommended the use of PCV7 (Prevnar), a 7-valent vaccine that was the first PCV, in all children aged <2 years (*8*). Use of PCV7 in children not only reduced the incidence of pneumococcal disease in those directly recommend for vaccination but also, through indirect effects, in older children and adults who were not recommended for vaccination (*9*). In 2010, PCV13 (Prevnar13), a 13-valent PCV, replaced PCV7 (no longer manufactured) and was recommended for routine use in all children aged 2–59 months (*10*). In 2012, ACIP recommended PCV13 in addition to PPSV23 for use in adults aged ≥19 years with an immunocompromising condition, functional or anatomic asplenia, a cerebrospinal fluid (CSF) leak, or a cochlear implant (*11*). In 2014, ACIP recommended routine use of PCV13 in series with PPSV23 for all adults aged ≥65 years (*12*). This recommendation was supported by a large, randomized controlled trial (RCT) among pneumococcal vaccine–naïve, community-dwelling adults aged ≥65 years in the Netherlands that demonstrated efficacy of PCV13 against noninvasive pneumococcal pneumonia (*13*). In June 2019 ACIP voted to no longer routinely recommend PCV13 for all adults aged ≥65 years and, instead, to recommend PCV13 on the basis of shared clinical decision-making for adults aged ≥65 years who do not have an immunocompromising condition, a CSF leak, or a cochlear implant (*14*). The rationale for this decision was based on additional years of data that found minimal changes in the incidence of pneumococcal disease preventable by PCV13 among adults after the PCV13 recommendation for all adults aged ≥65 years was implemented and on the historically low levels of PCV13-type disease attributed to the indirect effects of pediatric PCV13 use. Although anatomic or functional asplenia, chronic renal failure, and nephrotic syndrome might not be considered immunocompromising (*15*), these conditions are included under immunocompromising conditions for pneumococcal vaccine recommendations because of the similarities in the vaccine recommendations.

**FIGURE:**
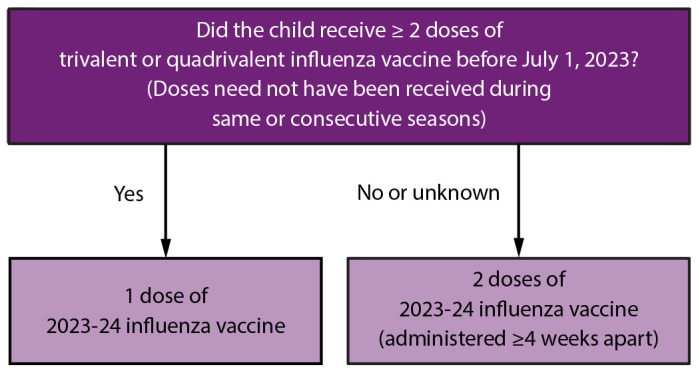
Serotypes contained in pneumococcal vaccines previously and currently used in the United States* **Abbreviations:** PCV7 = 7-valent pneumococcal conjugate vaccine; PCV13 = 13-valent pneumococcal conjugate vaccine; PCV15 = 15-valent pneumococcal conjugate vaccine; PCV20 = 20-valent pneumococcal conjugate vaccine; PPSV23 = 23-valent pneumococcal polysaccharide vaccine. * PCV7 is no longer manufactured.

**TABLE 1:** Licensed and available pneumococcal vaccines — United States, 2023

Vaccine product	Manufacturer	Trade name	Indications and age groups approved per package insert	No. of ACIP-recommended doses for adults	Year licensed or approved for adults
**Polysaccharide**					
PPSV23	Merck	Pneumovax23	PPSV23-type pneumococcal disease • Aged ≥50 years and aged ≥2 years who are at increased risk for pneumococcal disease	1–3 doses, depending on age and indications	1983
**Conjugate**					
PCV13	Pfizer	Prevnar13	PCV13-type IPD and PCV7-type otitis media • Aged 6 weeks through 5 years PCV13-type IPD • Aged 6–17 years PCV13-type IPD and pneumonia • Aged ≥18 years	1 dose	2011
PCV15	Merck	Vaxneuvance	PCV15-type IPD • Aged ≥6 weeks	1 dose	2021
PCV20	Pfizer	Prevnar20	PCV20-type IPD and PCV-type otitis • Aged 6 weeks through 5 years PCV20-type IPD • Aged 6–17 years PCV20-type IPD and pneumonia • Aged ≥18 years	1 dose	2021

**Abbreviations:** ACIP = Advisory Committee on Immunization Practices; IPD = invasive pneumococcal disease; PCV7 = 7-valent pneumococcal conjugate vaccine; PCV13 = 13-valent pneumococcal conjugate vaccine; PCV15 = 15-valent pneumococcal conjugate vaccine; PCV20 = 20-valent pneumococcal conjugate vaccine; PPSV23 = 23-valent pneumococcal polysaccharide vaccine.

### New ACIP Recommendations

Adults aged ≥19 years with an immunocompromising condition, a CSF leak, or a cochlear implant who have received both PCV13 and PPSV23 with incomplete vaccination status are recommended to complete their pneumococcal vaccine series by receiving either a single dose of PCV20 at an interval at least 5 years after the last pneumococcal vaccine dose or ≥1 dose of PPSV23 (Tables 2, 3, and 4). 

**TABLE 2:** Pneumococcal vaccine schedules for adults aged ≥65 years, by underlying conditions — Advisory Committee on Immunization Practices, United States, 2023

Vaccine received previously at any age	Any or no underlying condition	No specified immunocompromising condition,* CSF leak, or cochlear implant	Specified immunocompromising condition,* CSF leak, or cochlear implant
	Schedule option A (PCV20 available)	Schedule option B (PCV15 and PPSV23 available)	Schedule option B (PCV15 and PPSV23 available)
None/unknown^†^ or PCV7 only^§^	Administer a single dose of PCV20	Administer a single dose of PCV15, then after a ≥1 year interval since the PCV15 dose, administer a single dose of PPSV23	Administer a single dose of PCV15, then after ≥8 weeks since the PCV15 dose, administer a single dose of PPSV23
PPSV23 only^§^	Administer a single dose of PCV20 after a ≥1 year interval since the last PPSV23 dose	Administer a single dose of PCV15 after a ≥1 year interval since the last PPSV23 dose	Administer a single dose of PCV15 after a ≥1 year interval since the last PPSV23 dose
PCV13 only	Administer a single dose of PCV20 after a ≥1 year interval since the last PCV13 dose^¶^	Administer a single dose of PPSV23 after a ≥1 year interval since the last PCV13 dose**	Administer a single dose of PPSV23 after ≥8 weeks since the last PCV13 dose**
Both PCV13 and PPSV23 (any order of receipt) but has not yet received a dose of PPSV23 at age ≥65 years	Administer a single dose of PCV20 after a ≥5 year interval since the last PCV13 or PPSV23 dose^¶^	Administer a single dose of PPSV23 after a ≥1 year interval since the last PCV13 dose and a ≥5 year interval since the last PPSV23 dose**	Administer a single dose of PPSV23 after ≥8 weeks since the last PCV13 dose and ≥5 years since the last PPSV23 dose**
Both PCV13 and PPSV23 (any order), and the PPSV23 was administered at age ≥65 years	Together, with the patient, vaccine providers may choose to administer a single dose of PCV20 to adults aged ≥65 years who already have received PCV13 (but not PCV15 or PCV20) at any age and PPSV23 at age ≥65 years. The interval should be ≥5 years since the last PCV13 or PPSV23 dose.^¶,††^	N/A	N/A

**Abbreviations:** ACIP = Advisory Committee on Immunization Practices; CSF = cerebrospinal fluid; N/A = not applicable; PCV7 = 7-valent pneumococcal conjugate vaccine; PCV13 = 13-valent pneumococcal conjugate vaccine; PCV15 = 15-valent pneumococcal conjugate vaccine; PCV20 = 20-valent pneumococcal conjugate vaccine; PPSV23 = 23-valent pneumococcal polysaccharide vaccine.

* Chronic renal failure, congenital or acquired asplenia, congenital or acquired immunodeficiency (including B-[humoral] or T-lymphocyte deficiency, complement deficiencies [particularly C1, C2, C3, and C4 deficiencies], and phagocytic disorders [excluding chronic granulomatous disease]), generalized malignancy, HIV infection, Hodgkin disease, iatrogenic immunosuppression (including disease requiring treatment with immunosuppressive drugs such as long-term systemic corticosteroids and radiation therapy), leukemia, lymphoma, multiple myeloma, nephrotic syndrome, sickle cell disease or other hemoglobinopathies, and solid organ transplant. Excludes persons with a hematopoietic stem cell transplant (**Source:** Matanock A, Lee G, Gierke R, Kobayashi M, Leidner A, Pilishvili T. Use of 13-valent pneumococcal conjugate vaccine and 23-valent pneumococcal polysaccharide vaccine among adults aged ≥65 years: updated recommendations of the Advisory Committee on Immunization Practices. MMWR Wkly Rep 2019;68:1069–75).

^†^ New ACIP recommendation (October 2021) (**Source:** Kobayashi M, Farrar JL, Gierke R, et al. Use of 15-valent pneumococcal conjugate vaccine and 20-valent pneumococcal conjugate vaccine among U.S. adults: updated recommendations of the Advisory Committee on Immunization Practices—United States, 2022. MMWR Wkly Rep 2022;71:109–17).

^§^ CDC guidance implementation (**Source:** https://www.cdc.gov/vaccines/acip/meetings/downloads/slides-2022-10-19-20/04-Pneumococcal-Kobayashi-508.pdf).

^¶^ New ACIP recommendation (October 2022).

** **Source:** Matanock A, Lee G, Gierke R, Kobayashi M, Leidner A, Pilishvili T. Use of 13-valent pneumococcal conjugate vaccine and 23-valent pneumococcal polysaccharide vaccine among adults aged ≥65 years: updated recommendations of the Advisory Committee on Immunization Practices. MMWR Wkly Rep 2019;68:1069–75.

^††^ **Source**: CDC. Shared clinical decision-making: PCV20 vaccination for adults 65 years or older. Considerations for shared clinical decision-making might include the individual patient’s risk for pneumococcal disease due to underlying medical conditions, time since last pneumococcal vaccination, or risk for exposure to pneumococcal serotypes contained in PCV20. https://www.cdc.gov/vaccines/hcp/admin/downloads/job-aid-scdm-pcv20-508.pdf

**TABLE 3:** Pneumococcal vaccine schedules for adults aged 19–64 years with specified immunocompromising conditions* — Advisory Committee on Immunization Practices, United States, 2023

Vaccine received previously at any age	Schedule option A (PCV20 available)	Schedule option B (PCV15 and PPSV23 available)
None/unknown^†^ or PCV7 only^§^ at any age	Administer a single dose of PCV20	Administer a single dose of PCV15, then after a ≥8 week interval since the PCV15 dose, administer a single dose of PPSV23
PPSV23 only^§^	Administer a single dose of PCV20 after a ≥1 year interval since the last PPSV23 dose	Administer a single dose of PCV15 after a ≥1 year interval since the last PPSV23 dose
PCV13 only	Administer a single dose of PCV20 after a ≥1 year interval since the last PCV13 dose^¶^	Administer a single dose of PPSV23 after a ≥8 week interval since the last PCV13 dose. Administer a second PPSV23 dose after a ≥5 year interval since the last PPSV23 dose. Review the pneumococcal vaccine recommendations again when the patient turns age 65 years.**
PCV13 and 1 dose of PPSV23 (any order of receipt)	Administer a single dose of PCV20 after a ≥5 year interval since the last PCV13 or PPSV23 dose^¶^	Administer a single dose of PPSV23 after a ≥8 week interval since the last PCV13 dose and a ≥5 year interval since the last PPSV23 dose. Review the pneumococcal vaccine recommendations again when the patient turns age 65 years.**
PCV13 and 2 doses of PPSV23 (any order of receipt)	Administer a single dose of PCV20 after a ≥5 year interval since the last PCV13 or PPSV23 dose^¶^	Review the pneumococcal vaccine recommendations again when the patient turns age 65 years**

**Abbreviations:** ACIP = Advisory Committee on Immunization Practices; CSF = cerebrospinal fluid; PCV7 = 7-valent pneumococcal conjugate vaccine; PCV13 = 13-valent pneumococcal conjugate vaccine; PCV15 = 15-valent pneumococcal conjugate vaccine; PCV20 = 20-valent pneumococcal conjugate vaccine; PPSV23 = 23-valent pneumococcal polysaccharide vaccine.

* Chronic renal failure congenital or acquired asplenia, congenital or acquired immunodeficiency (including B-[humoral] or T-lymphocyte deficiency, complement deficiencies [particularly C1, C2, C3, and C4 deficiencies], and phagocytic disorders [excluding chronic granulomatous disease]), generalized malignancy, HIV infection, Hodgkin disease, iatrogenic immunosuppression (including disease requiring treatment with immunosuppressive drugs such as long-term systemic corticosteroids and radiation therapy), leukemia, lymphoma, multiple myeloma, nephrotic syndrome, sickle cell disease and other hemoglobinopathies, and solid organ transplant. Excludes persons with a hematopoietic stem cell transplant.

^†^ New ACIP recommendation (October 2021) (**Source:** Kobayashi M, Farrar JL, Gierke R, et al. Use of 15-valent pneumococcal conjugate vaccine and 20-valent pneumococcal conjugate vaccine among U.S. adults: updated recommendations of the Advisory Committee on Immunization Practices—United States, 2022. MMWR Wkly Rep 2022;71:109–17).

^§^ CDC guidance implementation (**Source:** https://www.cdc.gov/vaccines/acip/meetings/downloads/slides-2022-10-19-20/04-Pneumococcal-Kobayashi-508.pdf).

^¶^ New ACIP recommendation (October 2022).

** **Source:** Matanock A, Lee G, Gierke R, Kobayashi M, Leidner A, Pilishvili T. Use of 13-valent pneumococcal conjugate vaccine and 23-valent pneumococcal polysaccharide vaccine among adults aged ≥65 years: updated recommendations of the Advisory Committee on Immunization Practices. MMWR Wkly Rep 2019;68:1069–75.

**TABLE 4:** Pneumococcal vaccine schedules for adults aged 19–64 years with a cerebrospinal fluid leak or a cochlear implant — Advisory Committee on Immunization Practices, United States, 2023

Vaccine received previously	Schedule option A (PCV20 available)	Schedule option B (PCV15 and PPSV23 available)
None* or PCV7 only^†^ at any age	Administer a single dose of PCV20	Administer a single dose of PCV15, then after a ≥8 week interval since the PCV15 dose, administer a single dose of PPSV23
PPSV23 only^†^	Administer a single dose of PCV20 after a ≥1 year interval since the last PPSV23 dose	Administer a single dose of PCV15 after a ≥1 year interval since the last PPSV23 dose
PCV13 only	Administer a single dose of PCV20 after a ≥1 year interval since the last dose^§^	Administer a single dose of PPSV23 after a ≥8 week interval since the last PCV13 dose. Review the pneumococcal vaccine recommendations again when the patient turns age 65 years.^¶^
PCV13 and 1 dose of PPSV23	Administer a single dose of PCV20 after a ≥5 year interval since the last dose^§^	Review the pneumococcal vaccine recommendations again when the patient turns age 65 years^¶^

**Abbreviations:** ACIP = Advisory Committee on Immunization Practices; PCV7 = 7-valent pneumococcal conjugate vaccine; PCV13 = 13-valent pneumococcal conjugate vaccine; PCV15 = 15-valent pneumococcal conjugate vaccine; PCV20 = 20-valent pneumococcal conjugate vaccine; PPSV23 = 23-valent pneumococcal polysaccharide vaccine.

* New ACIP recommendation (October 2021) (**Source:** Kobayashi M, Farrar JL, Gierke R, et al. Use of 15-valent pneumococcal conjugate vaccine and 20-valent pneumococcal conjugate vaccine among U.S. adults: updated recommendations of the Advisory Committee on Immunization Practices—United States, 2022. MMWR Wkly Rep 2022;71:109–17).

^†^ CDC guidance implementation (**Source:** https://www.cdc.gov/vaccines/acip/meetings/downloads/slides-2022-10-19-20/04-Pneumococcal-Kobayashi-508.pdf).

^§^ New ACIP recommendation (October 2022).

^¶^ **Source:** Matanock A, Lee G, Gierke R, Kobayashi M, Leidner A, Pilishvili T. Use of 13-valent pneumococcal conjugate vaccine and 23-valent pneumococcal polysaccharide vaccine among adults aged ≥65 years: updated recommendations of the Advisory Committee on Immunization Practices. MMWR Wkly Rep 2019;68:1069–75.

— When a second PPSV23 is used instead of PCV20, it should be administered ≥8 weeks after the PCV13 dose and ≥5 years after the first PPSV23 dose for adults aged 19–64 years with an immunocompromising condition but not for adults with a CSF leak or a cochlear implant (Table 3). In addition, adults with an immunocompromising condition, a CSF leak, or a cochlear implant who have received both PCV13 and PPSV23 (no PCV20) but have not received a dose of PPSV23 at age ≥65 years are recommended to receive either PCV20 or a single final dose of PPSV23 at age ≥65 years and ≥5 years since the previous PPSV23 dose (*11*) (Tables 2, 3, and 4).

Shared clinical decision-making is recommended regarding PCV20 use for adults aged ≥65 years who have completed the recommended vaccine series with both PCV13 (at any age) and PPSV23 (which was administered at age ≥65 years) (Table 2). Unlike routine, catch-up, and risk-based recommendations, shared clinical decision-making vaccinations are not recommended for everyone in a particular age group or everyone in an identifiable risk group. Rather, shared clinical decision-making recommendations are individually based and guided by a decision process between the health care provider and the patient or guardian.

 Adults aged ≥19 years who have received PCV13 only are recommended to receive a single dose of PCV20 at an interval ≥1 year after receipt of the PCV13 dose or to receive ≥1 dose of PPSV23 to complete their pneumococcal vaccine series.

— When PPSV23 is used instead of PCV20, the minimum recommended interval between PCV13 and PPSV23 administration is ≥8 weeks for adults with an immunocompromising condition, a CSF leak, or a cochlear implant and ≥1 year for adults without these conditions (*11*) (Tables 2, 3, and 4). Either PCV20 or a second PPSV23 dose is recommended ≥5 years after the first PPSV23 dose for adults aged 19–64 years with specified immunocompromising conditions but not for adults with a CSF leak or a cochlear implant (Table 3). In addition, those who received both PCV13 (at any age) and PPSV23 (no PCV20) but have not received a dose of PPSV23 at age ≥65 years are recommended to receive either PCV20 or a single and final dose of PPSV23 at age ≥65 years and ≥5 years since the previous PPSV23 dose (*11*) (Table 2).

### Updated CDC Guidance for Implementation

Adults aged ≥19 years who have received PPSV23 only are recommended to receive a dose of either PCV20 or PCV15 at an interval ≥1 year after receipt of the last PPSV23 dose (Tables 2, 3, 4, and 5). This CDC guideline was presented to the ACIP but did not go to a full vote.

**TABLE 5:** Pneumococcal vaccine schedules for adults aged 19–64 years with a chronic medical condition* — Advisory Committee on Immunization Practices, United States, 2023

Vaccine received previously	Schedule option A (PCV20 available)	Schedule option B (PCV15 and PPSV23 available)
None^†^ or PCV7 only^§^ at any age	Administer a single dose of PCV20	Administer a single dose of PCV15, then after a ≥1 year interval since the last dose, administer a single dose of PPSV23
PPSV23 only^§^	Administer a single dose of PCV20 after a ≥1 year interval since the last PPSV23 dose	Administer a single dose of PCV15 after a ≥1 year interval since the last PPSV23 dose
PCV13 only^§,¶^	After a ≥1 year interval since the last dose, administer a single dose of PCV20	Administer a single dose of PPSV23 after a ≥1 year interval since the last PCV13 dose. Review the pneumococcal vaccine recommendations again when the patient turns age 65 years.
PCV13 and PPSV23^§,¶^	No vaccines are recommended at this time. Review the pneumococcal vaccine recommendations again when the patient turns age 65 years.	

**Abbreviations:** ACIP = Advisory Committee on Immunization Practices; PCV7 = 7-valent pneumococcal conjugate vaccine; PCV13 = 13-valent pneumococcal conjugate vaccine; PCV15 = 15-valent pneumococcal conjugate vaccine; PCV20 = 20-valent pneumococcal conjugate vaccine; PPSV23 = 23-valent pneumococcal polysaccharide vaccine.

* Alcoholism; chronic heart disease, including congestive heart failure and cardiomyopathies; chronic liver disease; chronic lung disease, including chronic obstructive pulmonary disease, emphysema, and asthma; cigarette smoking; or diabetes mellitus.

^†^ New ACIP recommendation (October 2021) (**Source:** Kobayashi M, Farrar JL, Gierke R, et al. Use of 15-valent pneumococcal conjugate vaccine and 20-valent pneumococcal conjugate vaccine among U.S. adults: updated recommendations of the Advisory Committee on Immunization Practices—United States, 2022. MMWR Wkly Rep 2022;71:109–17).

^§^ CDC guidance for implementation (**Source:** https://www.cdc.gov/vaccines/vpd/pneumo/downloads/pneumo-vaccine-timing.pdf).

^¶^ Adults aged 19–64 years with a chronic medical condition have not been recommended to receive PCV13 (**Source:** Matanock A, Lee G, Gierke R, Kobayashi M, Leidner A, Pilishvili T. Use of 13-valent pneumococcal conjugate vaccine and 23-valent pneumococcal polysaccharide vaccine among adults aged ≥65 years: updated recommendations of the Advisory Committee on Immunization Practices. MMWR Wkly Rep 2019;68:1069–75).

### New CDC Guidance for Implementation

Adults who, at any age, have received PCV7 only should follow the recommendations for adults who have not received a pneumococcal vaccine or whose vaccination history is unknown (Tables 2, 3, 4, and 5).

Adults aged ≥19 years who are hematopoietic stem cell transplant (HSCT) recipients are recommended to receive 4 doses of PCV20, starting 3–6 months after HSCT. Administer 3 doses of PCV20, 4 weeks apart starting 3–6 months after HSCT. Administer a fourth PCV20 dose ≥6 months after the third dose of PCV20 or ≥12 months after HSCT, whichever is later. If PCV20 is not available, 3 doses of PCV15 4 weeks apart, followed by a single dose of PPSV23 ≥1 year after HSCT, can be administered. For patients with chronic graft versus host disease (GVHD) who are receiving PCV15, a fourth dose of PCV15 can be administered in place of PPSV23 because these adults are less likely to respond to PPSV23. A patient’s clinical team is best informed to determine the appropriate timing of vaccination (Table 6).

**TABLE 6:** Pneumococcal vaccine schedules for adults aged ≥19 years who received hematopoietic stem cell transplant — Advisory Committee on Immunization Practices, United States, 2023

Vaccine received previously	Schedule option A (PCV20 available)	Schedule option B (PCV15 and PPSV23 available)
None after HSCT	Administer 3 doses of PCV20, 4 weeks apart starting 3–6 months after HSCT. Administer a fourth PCV20 dose ≥6 months after the third dose of PCV20, or ≥12 months after HSCT, whichever is later.*	Administer 3 doses of PCV15, 4 weeks apart starting 3–6 months after HSCT, followed by PPSV23 ≥12 months after HSCT if no chronic GVHD. If patient has chronic GVHD, administer a fourth dose of PCV15 in place of PPSV23.*
Received ≥1 dose of PCV13 or PCV15 after HSCT but has not received all 4 doses of pneumococcal vaccine	Administer ≥1 dose of PCV20 to complete the 4-dose PCV series. Administer the first 3 doses of PCV 4 weeks apart starting 3–6 months after HSCT. Administer a single dose of PCV20 ≥6 months after the third dose of PCV, or ≥12 months after HSCT, whichever is later.*	Administer a total of 3 doses of PCV, 4 weeks apart starting 3–6 months after HSCT, followed by PPSV23 ≥12 months after HSCT if no chronic GVHD. If patient has chronic GVHD, administer a fourth dose of PCV15 in place of PPSV23.*

**Abbreviations:** ACIP = Advisory Committee on Immunization Practices; GVHD = graft versus host disease; HSCT = hematopoietic stem cell transplant; PCV = pneumococcal conjugate vaccine; PCV13 = 13-valent pneumococcal conjugate vaccine; PCV15 = 15-valent pneumococcal conjugate vaccine; PCV20 = 20-valent pneumococcal conjugate vaccine; PPSV23 = 23-valent pneumococcal polysaccharide vaccine.

* A patient’s clinical team is best informed to determine the appropriate timing of vaccination.

These CDC guidelines were presented to the ACIP but did not go to a full vote.

## Methods

ACIP’s Pneumococcal Vaccines Work Group included voting members of ACIP; representatives of ACIP ex officio and liaison organizations; and scientific consultants with expertise in public health, vaccinology, medical specialties (e.g., family practice, geriatrics, infectious disease, internal medicine, and pediatrics), vaccine research, and assessments of vaccine efficacy and safety. The work group reviewed and discussed topics such as pneumococcal disease epidemiology, pneumococcal vaccine safety, immunogenicity of newly licensed vaccines, efficacy and effectiveness of existing pneumococcal vaccines, pneumococcal vaccine coverage, program feasibility, health equity, and cost-effectiveness. This report updates and replaces previously published ACIP recommendations for pneumococcal vaccine use in adults (*11*,*12*,*14*,*16*–*18*). The work group organized, evaluated, and discussed information to create the recommendations using the Evidence to Recommendation (EtR) framework and Grading of Recommendations Assessment, Development, and Evaluation (GRADE) (*19*). Data used for general clarifications to the recommendations were summarized based on findings from expert opinion of the Pneumococcal Vaccines Work Group and literature searches that were completed October 17, 2022, and updated before publication. All studies yielding pertinent information were deemed relevant for inclusion.

In 2010, ACIP adopted the GRADE approach to assess the certainty of evidence after a systematic review of literature (*20*). Subsequent ACIP recommendations made using the GRADE approach include the following: routine use of PCV13 and PPSV23 for immunocompromised adults (*11*); routine use of PCV13 in adults aged ≥65 years (*12*); shared clinical decision-making regarding use of PCV13 for adults aged ≥65 years who do not have an immunocompromising condition, a CSF leak, or a cochlear implant (*14*); use of PCV15 or PCV20 in adults aged 19–64 years with certain underlying medical conditions or other risk factors and all adults aged ≥65 years who have not previously received a pneumococcal conjugate vaccine or whose vaccination history is unknown (*16*); and the use of PCV20 in adults who previously received PCV13. GRADE evidence tables for these recommendations are available (https://www.cdc.gov/vaccines/acip/recs/grade/table-refs.html).

For new ACIP recommendations in this report, a systematic literature search that was performed in February 2021 was updated to review available evidence on the immunogenicity and safety of PCV15 and PCV20 among adults (*21*–*24*). GRADE was only used for evidence on PCV20 use in adults who previously received PCV13. To identify additional literature published during March 2021–March 2022, PubMed Medline, Embase, CINAHL, Scopus, Epistemonikos, and Cochrane library databases were searched using the previous search terms. Search results were supplemented by a search of ClinicalTrials.gov using the term “20-valent pneumococcal vaccine, adults” and PubMed Medline using the terms “PCV20” or “20-valent pneumococcal vaccine” (https://stacks.cdc.gov/view/cdc/131260). Unpublished data from trials identified through ClinicalTrials.gov but not yet publicly available were provided by the vaccine manufacturers. Evidence on safety and immunogenicity of PCV20 among adults who previously received a pneumococcal vaccine was assessed using GRADE (*19*).

In 2018, ACIP adopted the EtR framework to facilitate the assessment and ensure transparency of additional factors considered in developing vaccine recommendations, including target population values and preferences, resource use, equity, acceptability, and feasibility of implementation (*25*). The updated recommendation for PCV13 use among adults aged ≥65 years in 2019, for use of PCV15 or PCV20 in adults aged 19–64 years with certain underlying medical conditions or other risk factors or all adults aged ≥65 years, and for PCV20 use in adults who previously received PCV13, was further evaluated using the EtR framework (*26*). When data were not available, expert opinions from the Pneumococcal Vaccines Work Group members were sought (https://www.cdc.gov/vaccines/acip/recs/grade/etr.html).

To assess safety of PCV13 and PPSV23, a search was performed of published literature summarizing data from two postlicensure surveillance systems: the Vaccine Adverse Event Reporting System (VAERS) (https://www.cdc.gov/vaccinesafety/ensuringsafety/monitoring/vaers/index.html) and the Vaccine Safety Datalink (VSD) (https://www.cdc.gov/vaccinesafety/ensuringsafety/monitoring/vsd/index.html). VAERS is a national passive public health surveillance system operated by CDC and the Food and Drug Administration (FDA) and accepts reports from anyone, including health care professionals, vaccine manufacturers, patients, and caregivers (*27*). Health care professionals and patients are encouraged to report clinically important or unexpected adverse events, even if they are unsure whether the event is vaccine related. Medical records are requested routinely by VAERS staff members for reports of serious adverse events (SAEs) (e.g., death, life-threatening event, hospitalization, lasting disability after vaccination, or birth defect) (*27*). VAERS can identify rare adverse events and detect possible safety problems quickly, generating vaccine safety hypotheses to be evaluated by other sources; however, VAERS data typically cannot be used to determine whether a vaccine caused an adverse event.

VSD is a collaborative project among CDC, participating sites, and networks across the United States and uses electronic health data from participating sites to monitor and assess the safety of vaccines. VSD contains population-safety studies and can be used to assess hypotheses that arise from reviews of medical literature, reports to VAERS, changes in vaccination schedules, or introduction of new vaccines (*28*). For all pneumococcal vaccines, additional review of VAERS was conducted to assess safety data that were reported through August 31, 2022.

The ACIP Pneumococcal Vaccines Work Group met by teleconference regularly (usually once to twice per month). Experts external to the work group were invited to present current research and discuss published and unpublished data. The work group summarized and presented the evidence to ACIP to help formulate recommendations. ACIP votes were held when a new pneumococcal vaccine recommendation was under consideration (e.g., new age indications or dosing regimens for a vaccine), or when additional groups were identified as being at risk for pneumococcal disease. New ACIP recommendations in this report were discussed during the ACIP meeting on October 19, 2022. At that time ACIP members voted to approve a draft of the following adult pneumococcal vaccine recommendations: use of either a single dose of PCV20 or PPSV23 as previously recommended for adults with an immunocompromising condition, a CSF leak, or a cochlear implant who have started their pneumococcal vaccine series with PCV13 but have not received all recommended PPSV23 doses; shared clinical decision-making regarding PCV20 use for adults aged ≥65 years who have completed their recommended vaccine series with both PCV13 and PPSV23; and use of either a single dose of PCV20 or PPSV23 as previously recommended for adults who have received PCV13 only. These recommendations were based on pre–COVID-19 pandemic pneumococcal disease burden data because the incidence of pneumococcal disease has been dynamic since the pandemic onset (i.e., a reduction of invasive pneumococcal disease early in the pandemic, with reports of increases after countries relaxed their nonpharmaceutical interventions) (*29*,*30*). In addition, work group and ACIP members reviewed and commented on a draft of updated and new CDC guidance for implementation for adults who have received PPSV23 only, adults who have received PCV7 only, and adults who are HSCT recipients. The guidelines for implementation were presented during the October 19 meeting with supporting data but were not subjected to a vote because the intent was only to provide guidance on how to apply the new ACIP recommendations for specific populations.

## Transmission of Pneumococcus

Pneumococcus can colonize the upper respiratory tract of humans, and prevalence of pneumococcal colonization typically increases during the first 2 years of life and decreases thereafter (*2*,*31*). Pneumococci are transmitted primarily through respiratory droplets from pneumococcal carriers, and young children are a common reservoir of pneumococci in the community because of a higher frequency of pneumococcal colonization (*2*). Among older adults, those who live in nursing homes, smoke cigarettes, or have regular contact with children aged <6 years have higher pneumococcal carriage than those without these risk factors (*32*). Pneumococcal colonization is a precursor to disease, and pneumococci can spread beyond the upper respiratory tract and cause disease ranging from acute otitis media through local spread from the upper respiratory tract, to pneumonia through aspiration, or to IPD such as bacteremia or meningitis through bacteremia (*1*). Inflammatory conditions in the upper respiratory tract, including coinfection with respiratory viruses in particular, can increase pneumococcal density in the upper respiratory tract and facilitate transmission and microaspiration to the lungs (*1*). Reductions in the incidence of pneumococcal disease were observed globally early in the COVID-19 pandemic (*33*,*34*). However, certain studies in children reported that pneumococcal carriage did not change during this period, and the reduction in pneumococcal disease has been attributed to reduced circulation of respiratory viruses (e.g., influenza virus and respiratory syncytial virus) because of the nonpharmaceutical interventions that were implemented (*34*–*36*).

## Epidemiology of Pneumococcal Disease in U.S. Adults

In adults, the risk for pneumococcal disease increases with increasing age. Pneumococcal pneumonia is the most common form of pneumococcal disease in adults and is estimated to account for approximately 10% of hospitalized community-acquired pneumonia (CAP) cases (*37*). According to commercial claims and encounter data, in 2014, an estimated 680 (adults aged 18–49 years) to 9,570 (adults aged ≥85 years) all-cause pneumonia cases per 100,000 person-years resulted in health care use; during 2008–2014 the proportion of all-cause pneumonia cases that resulted in hospitalization was higher in older age groups, ranging from 8.5% among adults aged 18–49 years to 38.6% among adults aged ≥85 years (*38*). When limited to studies that collected data during 2010–2016, a systematic review of the literature on all-cause CAP hospitalization in the United States indicated that the estimated incidence ranges were 126–422 per 100,000 adults aged <65 years and 847–3,365 per 100,000 adults aged ≥65 years (*39*). In a multistate surveillance study of hospitalized nonbacteremic pneumococcal pneumonia (NBPP) patients, the estimated incidence of pneumococcal pneumonia was 12 per 100,000 adults aged 18–49 years and 105 per 100,000 adults aged ≥65 years in 2017 (*40*). An assessment of adults hospitalized with clinical and radiographic evidence of pneumonia in Nashville, Tennessee, and Atlanta, Georgia, reported that the incidence of pneumococcal pneumonia hospitalizations ranged from 43 to 53 per 100,000 population (*41*).

Data on pneumococcal serotype distribution among noninvasive pneumococcal pneumonia cases are limited; respiratory culture is performed infrequently, and available data mostly rely on serotype-specific urinary antigen detection (SSUAD) tests that are only available for research use (*37*). In a prospective multisite study of U.S. adults hospitalized with CAP during October 2013–September 2016 that used either pneumococcal isolates from culture or SSUAD to determine pneumococcal serotype, the proportion of hospitalized all-cause pneumonia cases due to PCV13 serotypes was 4.6% (559 of 12,055), the proportion attributable to the two serotypes included in PCV15 and not in PCV13 (22F and 33F) was an additional 1.4% (166 of 12,055), and the proportion of the seven serotypes included in PCV20 and not in PCV13 (serotypes 8, 10A, 11A, 12F, 15B/15C,* 22F, and 33F) was 3.3% (400 of 12,055) (*37*). IPD incidence and serotype distribution data are available from CDC’s Active Bacterial Core surveillance (ABCs), an active laboratory- and population-based system across 10 sites in the United States with a catchment population of approximately 34 million persons for IPD surveillance (*42*). In 2019, IPD incidence ranged from 2.3 per 100,000 population among adults aged 18–34 years to 37.8 among adults aged ≥85 years. During 2018–2019, among adults aged ≥65 years, the proportions of IPD due to PCV13, PCV15, PCV20, and PPSV23 serotypes were 27%, 42%, 54%, and 62%, respectively.

After PCV was recommended for use in children, the incidence of PCV-type pneumococcal disease decreased in adults who were not recommended to receive the vaccine, including those with underlying medical conditions who are at increased risk for pneumococcal disease (*43*). However, IPD incidence remained stable during 2014–2019, with the most common remaining PCV13-type serotype being serotype 3 (62% of PCV13 serotypes among adults aged ≥65 years during 2018) (*44*,*45*). Among U.S. adults aged ≥65 years hospitalized for CAP, the proportion of PCV13-type serotypes assessed by use of SSUAD or pneumococcal isolates from culture declined from 5.3% during 2013–2014 to 3.4% during 2015–2016. In contrast, among adults aged 18–64 years who did not have a recommendation for PCV13 vaccination, the proportion of all-cause CAP cases with PCV13-type serotypes did not change. Of note, the number of participants in the latter groups was smaller compared with adults aged ≥65 years (6,347 aged ≥65 years, 2,976 aged 18–64 years at increased risk, and 868 aged 18–64 years with low-risk conditions); therefore, the serotype distribution might be less stable (*46*).

In 2020, a decline in IPD incidence was observed globally, and the decline is thought to be a result of the introduction of nonpharmaceutical interventions to contain the COVID-19 pandemic (e.g., mask wearing, staying at home, and restrictions on indoor gathering), which also likely reduced transmission of *S. pneumoniae* (*33*). In the United States, compared with the expected IPD rates on the basis of data from January 2014 through February 2020, observed IPD rates during March–December 2020 were 58% lower among all age groups and 68% lower among adults aged ≥65 years (*47*). However, more recent data from Europe and the United States have indicated an increase of IPD cases after countries relaxed their nonpharmaceutical interventions (*29*,*30*,*45*) (Active Bacterial Core surveillance, CDC, unpublished data, November 8, 2022).

### Pneumococcal Vaccine Coverage

PCV13 vaccination coverage increased among adults aged ≥65 years after the ACIP recommended routine use of PCV13 for this population in 2014 (*12*). Among Medicare Part A/B beneficiaries aged ≥65 years, the proportion who received at least 1 dose of PCV13 increased from 3% in 2014 to 49% in 2019, and the proportion who received both PCV13 and PPSV23 increased from 2% in 2014 to 32% in 2019 (*48*). Pneumococcal vaccine coverage among adults aged 19–64 years recommended to receive pneumococcal vaccine because of risk factors for pneumococcal disease has been low (see Groups at Increased Risk for Pneumococcal Disease). In 2018, findings from the National Health Interview Survey indicated that 23% of adults aged 19–64 years who are at increased risk for pneumococcal disease had ever received a pneumococcal vaccine (*49*). Administrative claims data among adults aged 19–64 years with newly diagnosed medical conditions considered to be risk factors for pneumococcal disease demonstrated that overall pneumococcal vaccine coverage was 14% during the follow-up period (1–7 years since diagnosis) and varied among conditions; those with a diagnosis of HIV/AIDS had the highest pneumococcal vaccination coverage (48%) compared with those with a diagnosis of alcohol dependence (6%) (*50*). Vaccine coverage varies by sociodemographic characteristics, and Hispanic persons consistently have had lower pneumococcal vaccine coverage than other racial or ethnic groups, both among adults aged ≥65 years and younger adults with risk factors (*48*,*49*,*51*).

### Groups at Increased Risk for Pneumococcal Disease

In adults, the risk for pneumococcal infection increases with increasing age. In addition, adults with certain underlying medical conditions or risk factors have increased risk for pneumococcal disease compared with adults without these conditions and thus have risk-based pneumococcal vaccine recommendations regardless of age (Table 7).

**TABLE 7:** Recommendations for use of PCV15 or PCV20 in pneumococcal conjugate vaccine–naïve adults aged ≥19 years — Advisory Committee on Immunization Practices, United States, 2023

Medical indication group	Specific underlying medical condition	Age group, yrs	
		19–64	≥65
None	None	None	1 dose of PCV20 alone, or 1 dose of PCV15 followed by a dose of PPSV23 ≥1 year later*
Underlying medical conditions or other risk factors	Alcoholism Chronic heart disease^†^ Chronic liver disease Chronic lung disease^§^ Chronic renal failure^¶^ Cigarette smoking Cochlear implant Congenital or acquired asplenia^¶^ Congenital or acquired immunodeficiencies^¶,^** CSF leak Diabetes mellitus Generalized malignancy^¶^ HIV infection Hodgkin disease^¶^ Iatrogenic immunosuppression^¶,††^ Leukemia^¶^ Lymphoma^¶^ Multiple myeloma^¶^ Nephrotic syndrome^¶^ Sickle cell disease or other hemoglobinopathies^¶^ Solid organ transplant^¶^	1 dose of PCV20 alone or 1 dose of PCV15 followed by a dose of PPSV23 ≥1 year later*	1 dose of PCV20 alone or 1 dose of PCV15 followed by a dose of PPSV23 ≥1 year later*

**Abbreviations**: CSF = cerebrospinal fluid; PCV15 = 15-valent pneumococcal conjugate vaccine; PCV20 = 20-valent pneumococcal conjugate vaccine; PPSV23 = 23-valent pneumococcal polysaccharide vaccine.

* Adults with immunocompromising conditions, a CSF leak, or a cochlear implant might benefit from shorter intervals (e.g., ≥8 weeks). These vaccine doses do not need to be repeated at age ≥65 years if administered at age <65 years.

^†^ Includes congestive heart failure and cardiomyopathies.

^§^ Includes chronic obstructive pulmonary disease, emphysema, and asthma.

^¶^ Indicates immunocompromising conditions

** Includes B- (humoral) or T-lymphocyte deficiency, complement deficiencies (particularly C1, C2, C3, and C4 deficiencies), and phagocytic disorders (excluding chronic granulomatous disease).

^††^ Diseases requiring treatment with immunosuppressive drugs, including long-term systemic corticosteroids and radiation therapy.

#### Persons With Chronic Heart Disease

Persons with chronic heart disease (including congestive heart failure and cardiovascular and valve diseases) are reported to have as high as 3.3 times the odds for CAP and 9.9 times the odds for IPD compared with those without chronic heart disease (*52*). Among adults with chronic heart disease, the risk for CAP is higher, especially among those with severe congestive heart failure (*52*). Pneumococcal vaccination has been associated with a 22% reduction in all-cause mortality among adults with cardiovascular disease or very high cardiovascular risk (*53*).

#### Persons Who Smoke Cigarettes

Current smoking status has been associated with increased risk for pneumococcal carriage among older adults (*32*), and the risk for CAP increases with increasing number of pack-years (*52*). The reported risk for IPD among smokers has been more variable in the published literature (*52*), although an analysis using laboratory- and population-based IPD surveillance data in the United States indicated that tobacco smoking (defined as any smoking within the past year) had 2.8–4.1 times the risk for IPD compared with adults without underlying conditions that increase the risk for pneumococcal disease (*43*). Potential explanations for this increased risk include the increased risk for pneumococcal carriage and increased susceptibility to respiratory viral infections (*32*).

#### Persons With Chronic Lung Disease

Persons with chronic obstructive pulmonary disease (COPD) have higher risk for being hospitalized with CAP and IPD than persons with other chronic lung disease (*52*,*54*–*56*). Among adults aged ≥40 years hospitalized with CAP, those with COPD had 18 times the risk for CAP compared with those without COPD (*57*). The increased risk among persons with COPD might be because of increased inhaled corticosteroid use, reduced airway defense mechanisms, and an association with smoking (*52*,*54*). Previously, asthma was not considered to increase the risk for pneumococcal disease unless it occurred with chronic bronchitis, emphysema, or long-term systemic corticosteroid use (*58*). However, in 2010, ACIP updated the recommendations to add asthma as an indication for pneumococcal vaccination on the basis of data indicating that history of asthma was associated with increased risk for IPD even after adjusting for underlying conditions (*59*,*60*).

#### Persons With Diabetes Mellitus

Persons with diabetes (either type 1 or type 2) have been reported to have up to 1.4 times the risk for CAP compared with those without diabetes (*52*) and 1.4–5.9 times the risk for IPD compared with those without other risk conditions (*43*,*52*). The relative risk (RR) for pneumococcal disease is higher among younger adults with diabetes (*52*). For example, a population-based case-control study in Denmark found that the RR for pneumonia-related hospitalization in adults aged <40 years with diabetes compared with those without diabetes was 3.2, and the RR decreased with increasing age to 1.1 in adults aged ≥80 years (*61*). In addition to the comorbidities associated with diabetes that can increase the risk for pneumococcal disease, an impaired immune system that can occur as a result of persistent hyperglycemia has been implicated as a cause of increased risk for infection (*52*,*62*,*63*).

#### Persons With Excessive Alcohol Use

Excessive alcohol use has been associated with impaired host immune response against bacterial pneumonia (*64*–*66*) and has been associated with increased risk for CAP with a dose-response relation (*67*,*68*). In one systematic review, the risk for CAP among adults with clinically defined alcohol use disorder was 8.2 times higher compared with those without alcohol use disorder (*68*). IPD risk has been reported to be 2.3–7.7 times higher compared with adults without underlying conditions (*43*,*69*).

#### Persons With Chronic Liver Disease

The risks for morbidity and mortality from bacterial infections are high among adults with chronic liver disease, especially those with cirrhosis (*70*,*71*). In a retrospective cohort of health care claims repositories, the incidence of all-cause pneumonia in adults with chronic liver disease was 4.1–5.6 times higher, the risk for pneumococcal pneumonia was 4.3–6.4 times higher, and the incidence of IPD was 6.4–10.2 times higher compared with adults without underlying health conditions (*69*). In another study using IPD surveillance data, IPD incidence was 3.8–15.4 times higher compared with adults without underlying conditions (*43*).

#### Persons With Cochlear Implants

In 2002, increased risk for bacterial meningitis was reported among persons who received cochlear implants, with pneumococcus being the most common causative pathogen (*72*). As a result, CDC updated the pneumococcal vaccine recommendations to include cochlear implant recipients among those at increased risk for pneumococcal disease (*73*). This increased risk for meningitis was observed >2 years postimplementation of the cochlear implant (*72*,*74*). Health care claims data indicated that adults aged ≥65 years with cochlear implants have 10.5 times the risk for IPD compared with those without any underlying conditions (*69*). This study found that adults with cochlear implants also have increased risk for all-cause and pneumococcal pneumonia compared with adults without underlying conditions (*69*).

#### Persons With a CSF Leak

Persons with a CSF leak from various causes (e.g., congenital lesions, skull fractures, or neurosurgical procedures) are at increased risk for recurrent bacterial meningitis because of anatomic deficits in the natural barriers of the brain (*75*,*76*). In a cohort of adults with community-acquired bacterial meningitis, 59% of adults with a CSF leak had a previous episode of meningitis (*75*). A study among Kaiser Permanente Northern California (KPNC) members demonstrated that adults with a CSF leak had 6.4 times the risk for IPD compared with the risk among all members (*77*).

#### Persons With Functional or Anatomic Asplenia

The spleen plays an important role in clearing encapsulated bacteria (e.g., pneumococcus) from the bloodstream, because encapsulated bacteria evades opsonization and consequently direct recognition by macrophages (*78*). Therefore, adults with functional or anatomic asplenia (e.g., sickle cell disease or splenectomy) are at increased risk for pneumococcal infection. Health care claims data indicated that adults with functional or anatomic asplenia had 8.5–18.2 times increased risk for all-cause pneumonia and 14.0–32.6 times increased risk for IPD compared with adults without any underlying conditions (*69*).

#### Persons With Chronic Kidney Disease

Persons with chronic kidney disease (CKD), particularly those with nephrotic syndrome and end-stage renal disease (ESRD), have an altered immune system (*79*,*80*) and are at increased risk for pneumococcal disease. A study among KPNC members demonstrated that adults with stage 3 or 4 CKD (glomerular filtration rate [GFR] of 15–59 mL/min) had 1.2 times the risk for IPD, and adults with ESRD (GFR of <15 mL/min or on dialysis) had 3.7 times the risk for IPD compared with KPNC members overall (*77*). The 2012 Kidney Disease: Improving Global Outcomes clinical practice guideline recommends pneumococcal vaccination for adults with stages 4 and 5 CKD (*81*). A study that compared immunogenicity of PPSV23 in adults with CKD and healthy adults found that adults with CKD had significantly lower immune response to PPSV23 (*82*). Another study reported rapid decline of antibody levels after PPSV23 was administered in children and young adults with CKD (*83*). Persons on dialysis are known to have rapid decline of antibody concentrations, which could be associated with impaired adaptive immune system, as well as gradual removal of a proportion of the serum antibody through dialysis (*83*,*84*).

#### Persons With Primary or Secondary Immunocompromising Conditions

Persons with primary (congenital) immunodeficiency (i.e., conditions that cause B- or T-cell deficiency, complement deficiency, or phagocytic disorders) or secondary (acquired) immunodeficiency (e.g., HIV infection, hematopoietic malignancy, and treatment with radiation or immunosuppressive medications) are at increased risk for infection or severe manifestations from vaccine-preventable diseases including pneumococcal disease (*43*,*69*,*77*,*85*,*86*). In these adults, immunologic response to pneumococcal vaccines, especially response to PPSV23, is considered to be diminished and short lived (*87*–*90*). Additional information about the degree of immune suppression associated with different medical conditions and treatments is available through ACIP’s general best practices for vaccination of persons with altered immunocompetence (*15*), CDC’s Yellow Book (*91*), and the 2013 IDSA Clinical Practice Guideline for Vaccination of the Immunocompromised Host (*92*). Adults who are HSCT recipients are addressed separately.

#### Persons With Other Medical Conditions, Occupational or Environmental Exposures, or Living Conditions

Other medical conditions, occupational or environmental exposures, or living conditions have been associated with increased risk for pneumococcal disease in certain reports but are not part of the risk-based pneumococcal vaccine recommendations. Persons in these groups include adults with obesity (*93*,*94*), adults experiencing homelessness (*95*–*97*), and adults who work as welders (*98*–*100*).

### Management in Pneumococcal Disease Outbreak Settings

Outbreaks of pneumococcal disease have been reported in congregate settings, such as hospital wards (*101*), military training facilities (*102*,*103*), correctional facilities (*102**,**103*), and long-term care facilities (*104*–*106*). CDC does not have recommendations on the use of postexposure antimicrobial chemoprophylaxis for close contacts of a patient with pneumococcal disease or on the use of pneumococcal vaccines during an outbreak. However, antimicrobial chemoprophylaxis and vaccination frequently have been used to interrupt transmission of pneumococcus during outbreaks, in addition to infection prevention and control measures such as reinforcing hand and respiratory hygiene, patient isolation or patient cohorting, restriction of patient movements, or closure of affected wards or units (*107*–*109*).

Use of antimicrobial chemoprophylaxis can eliminate pneumococcal carriage and interrupt ongoing transmission among susceptible persons during an outbreak (*107*). A recent systematic review found that selection of antibiotics and the regimen used to control outbreaks in closed settings have been variable, which might depend on the antibiotic susceptibility pattern (*108*). Use of azithromycin, benzathine penicillin G, amoxicillin, or levofloxacin has been reported in recent outbreaks to prevent additional cases (*102*,*107*). If an exposed person has received vaccines that contain the serotype of the circulating pneumococcus, they are expected to have longer-term protection against pneumococcal disease than if they were to receive antimicrobial chemoprophylaxis (*107*). Specifically, PCVs have advantages over PPSV23 because these vaccines can induce high levels of serotype-specific immunoglobulin G (IgG) to help protect vaccinated persons from vaccine-type pneumococcal carriage (*110*,*111*). Outbreaks of vaccine-serotype pneumococcal disease have been reported in settings where pneumococcal vaccine coverage is low (*105*,*106*,*112*). Therefore, outbreaks provide an opportunity to assess vaccination history and ensure that both persons with pneumococcal disease and exposed persons are up to date with their recommended vaccinations.

## Pneumococcal Vaccines and Administration

One PPSV and three PCVs are licensed and available for use in the United States (Table 1). These vaccines are PPSV23 (Pneumovax23), a 23-valent pneumococcal polysaccharide vaccine; PCV13 (Prevnar13), a 13-valent pneumococcal conjugate vaccine; PCV15 (Vaxneuvance), 15-valent pneumococcal conjugate vaccine; and PCV20 (Prevnar20), a 20-valent pneumococcal conjugate vaccine. The PPSV and PCV vaccines induce immune responses in different ways. Studies in mice have found that the polysaccharide vaccine induces a T-cell independent immune response and stimulates immediate B-cell responses (*113*). As a result, B-cells differentiate to plasma cells that produce antibodies. However, a T-cell independent immune response does not result in creation of serotype-specific memory B-cells (*114*). On the other hand, conjugate vaccines induce a T-cell dependent response. The polysaccharide antigen binds to the B-cells, and the peptides from the carrier protein are presented to carrier-peptide–specific helper T-cells. These helper T-cells enhance the immune response by the B-cells, and memory B-cells also are created (*114*,*115*). All PCVs use CRM197 (genetically detoxified diphtheria toxin) as a carrier protein (*116*–*118*).

PCV15 and PCV20 are administered intramuscularly at a dose of 0.5 mL. If PCV13 is used in situations where PCV15 or PCV20 is not easily accessible, it is administered intramuscularly at a dose of 0.5 mL. PPSV23 is administered either intramuscularly or subcutaneously at a dose of 0.5 mL. In a small RCT among adults aged ≥55 years, no significant differences were observed in the immunogenicity (measured as IgG geometric mean concentration [GMC] pre- and 1-month post PPSV23 vaccination for serotypes 3, 4, and 6B) by route of administration (*119*). Local adverse events were more common among those who received PPSV23 subcutaneously (18.9% [nine of 127] versus 7.1% [24 of 127]), whereas the frequency of systemic adverse events was the same in both groups (6.3% [eight of 127]) (*119*). All three pneumococcal vaccines (i.e., PCV15, PCV20, and PPSV23) are available in a single-dose, prefilled syringe. PPSV23 also is available in a single-dose vial. Additional information on pneumococcal vaccine administration is available in the package inserts (*116*,*117*,*120*).

### Vaccine Efficacy and Effectiveness

Vaccine efficacy refers to the percentage by which the rate of the target disease among those who are vaccinated is reduced compared with the rate among unvaccinated persons in the context of a placebo controlled randomized trial. Vaccine effectiveness (VE) measures the same percent reduction in the rate of disease as efficacy, but in the context of routine, real-world use of the vaccine through observational studies. Vaccine efficacy and effectiveness data are currently not available for PCV15 or PCV20.

### 23-Valent Pneumococcal Polysaccharide Vaccine (PPSV23)

PPSV23 contains pneumococcal polysaccharide serotypes 1, 2, 3, 4, 5, 6B, 7F, 8, 9N, 9V, 10A, 11A, 12F, 14, 15B, 17F, 18C, 19F, 19A, 20, 22F, 23F, and 33F (Figure). Each 0.5-mL dose contains 25 *μ*g of each polysaccharide type in isotonic saline solution containing 0.25% phenol as a preservative (*120*).

#### Clinical Efficacy and Effectiveness in Adults

Multiple RCTs and observational studies have reported that PPSV23 is protective against IPD. A meta-analysis of RCTs reported a pooled vaccine efficacy of 63% (95% CI = 10%–92%) against IPD due to any serotype (*121*); the RCTs included in this meta-analysis did not report PPSV23 efficacy against vaccine-type (VT) IPD (VT-IPD) (*122*–*125*). A recent meta-analysis of observational studies reported a pooled VE estimate against VT-IPD of 45% (95% CI = 37%–51%), which is lower than the estimate against all-cause IPD from RCTs, even when limiting to VE estimates ≤5 years since PPSV23 vaccination (*126*). The lower pooled VE estimate might be because of the presence of adults with immunocompromising conditions who were excluded upon enrollment from most of the RCTs. A pooled VE estimate against VT-IPD in adults without immunocompromising conditions was 60% (95% CI = 47%–69%) (*126*).

Estimates of the efficacy and effectiveness of PPSV23 against pneumococcal pneumonia have been more variable across studies. A pooled estimate of RCTs evaluating efficacy of PPSV23 against pneumococcal pneumonia was 35% (95% CI = −62% to 65%), but when results were limited to two RCTs deemed to have low risk for bias only, the pooled estimate was 64% (95% CI = 35%–80%) (*121*). These RCTs did not report efficacy against VT-pneumococcal pneumonia. Observational studies have reported VE estimates ranging from none or not significant (*127*,*128*) to moderate (40%–50%) (*129*,*130*) against pneumococcal pneumonia. The heterogeneity in the estimates reported in these observational studies is likely because of differences in study design, including how pneumococcal pneumonia cases were ascertained, study settings (community based versus hospital based), proportion of adults with underlying conditions, or age distribution. The pooled VE estimate against VT-pneumococcal pneumonia from five observational studies was not significant (VE = 18%; 95% CI = −4% to 35%) (*126*).

#### Clinical Efficacy and Effectiveness in Adults With Immunocompromising Conditions

One RCT among Ugandan adults living with HIV infection conducted during 1995–1998 observed no protection against IPD due to all serotypes (VE = −48%; 95% CI = −232% to 35%) or VT-IPD (VE = −114%; 95% CI = −431% to 14%). Of note, this study was conducted before highly active antiretroviral therapy was widely available, >10% of the vaccinated and placebo groups were lost to follow-up, and 28% of remaining participants in each group had died by the end of the study period (*131*). Observational studies have demonstrated mixed results regarding PPSV23 VE against IPD among adults with HIV infection (*132*–*136*). Three indirect cohort studies reported VT-IPD effectiveness by risk groups in the same study (*137*–*139*). Typically, adults with immunocompromising conditions had lower VE than adults without underlying medical conditions, and VE in older adults (aged ≥65 years) often was not significant when ≥2 years had passed since PPSV23 vaccination (*137*,*138*).

#### Duration of Protection

Data on duration of protection after PPSV23 vaccination are only available through observational studies. In a large, indirect cohort study from England and Wales, VE of PPSV23 against VT-IPD declined significantly with time since vaccination from 48% (95% CI = 32%–60%) among those vaccinated ≤2 years ago to 15% (95% CI = −3% to 30%) among those vaccinated ≥5 years ago (p<0.001) (*137*). In another study from England and Wales, VE of PPSV23 against VT-IPD declined from 41% (95% CI = 23%–54%) among those vaccinated ≤2 years ago to 34% (95% CI = 16%–48%) among those vaccinated 2–4 years ago and to 23% (95% CI = 12%–32%) among those vaccinated ≥5 years ago (*138*). Although this difference in VE by time since vaccination was not statistically significant (p = 0.13), a spline model indicated a decline in VE from approximately 50% to a plateau of 20%–25% after 5 years. Both studies found a declining trend in VE against VT-IPD regardless of age at vaccination. In the three studies on the duration of protection of PPSV23 against CAP, diagnostic criteria for determining pneumococcal CAP varied (*129*,*140*,*141*). Nevertheless, a declining trend in VE against CAP was observed with increasing time since vaccination in all identified studies.

#### Repeat PPSV23 Administration

Repeat administration of bacterial polysaccharides vaccines might induce immune tolerance or hyporesponsiveness to vaccine antigens (*142*). In the RCT of PPSV23 among Ugandan adults with HIV infection, researchers hypothesized that the increased risk for pneumococcal disease that was observed among persons who received PPSV23 compared with those who had received placebo was due to destruction of polysaccharide-responsive B-cell clones (*131*).

A systematic review of immunogenicity studies investigating repeat administration of PPSV23 found that revaccination with PPSV23 might induce a lower immune response than initial vaccination in the short-term (i.e., when measured ≤2 months after each vaccination); however, these differences were not statistically significant. When immune responses measured >2 years after vaccination were compared, antibody levels against certain serotypes were higher after revaccination with PPSV23 compared with initial vaccination (*143*). In a more recent systematic review of immunogenicity studies, immune responses were lower with repeat PPSV23 than with the initial vaccination for certain serotypes when the interval between PPSV23 doses was <5 years, whereas no evidence of hyporesponsiveness was noted in studies that used an interval of ≥5 years, an interval that has been recommended for adults with indications for ≥2 PPSV23 doses (*144*). Because no clinical trials are investigating multiple-dose PPSV23 regimens, the clinical relevance of hyporesponsiveness on pneumococcal disease is unclear.

#### Safety

A postlicensure review of PPSV23 safety was conducted using VAERS data during 1990–2013, which included 25,168 reports of adverse events after PPSV23 receipt. No safety signals or new or unexpected adverse events were identified in this study (*145*).

An updated search of VAERS data during January 1, 2014–August 31, 2022 found 24,926 reports; 23,763 (95.3%) were nonserious, 20,737 (83.2%) were in adults aged ≥19 years, and 15,550 (62.4%) were in females (P Moro, MD, CDC, personal communication, September 7, 2022). When PPSV23 was administered alone, injection site erythema (21.1%), injection site swelling (19.3%), and injection site pain (19.3%) were the most commonly reported adverse events (on the basis of MedDRA preferred terms) overall. These rates are similar to commonly reported adverse events across all vaccines administered to adults: injection site erythema (27.1%), erythema (24.7%), injection site swelling (23.6%), and injection site pain (23.3%). Of 35 deaths reported, 30 were adults, two were children, and three were of unknown age. Clinical review of these death reports did not reveal any pattern suggestive of a causal association with PPSV23. Half of the 26 reports of anaphylaxis after PPSV23 receipt were after receipt of PPSV23 alone. The VAERS reporting rate for 26 reports of anaphylaxis was 1 case per million PPSV23 doses distributed in the United States during the review period (January 1, 2014–August 31, 2022), similar to the incidence of vaccine-triggered anaphylaxis (0.8 per million doses administered) across all vaccines (*146*). Data mining analysis did not reveal disproportional reporting of any new or unexpected adverse events after PPSV23 receipt.

### 13-Valent Pneumococcal Conjugate Vaccine (PCV13)

PCV13 contains pneumococcal polysaccharide serotypes 1, 3, 4, 5, 6A, 6B, 7F, 9V, 14, 18C, 19A, 19F, and 23F, conjugated to CRM197, a nontoxic form of diphtheria toxin (Figure). Each 0.5-mL dose of vaccine contains approximately 2.2 *μ*g of all serotypes except for 4.4 *μ*g of 6B saccharides, 34 *μ*g of CRM197 carrier protein, 100 *μ*g of polysorbate 80, 295 *μ*g of succinate buffer, and 125 *μ*g of aluminum as aluminum phosphate adjuvant (*118*). In 2021, ACIP updated the PCV13 recommendations to replace PCV13 with PCV15 or PCV20 for adults who have not received a pneumococcal conjugate vaccine (*16*).

#### Clinical Efficacy and Effectiveness in Adults With and Without Immunocompromising Conditions

Studies evaluating PCV13 efficacy or effectiveness in adults are limited. A RCT among 84,496 Dutch pneumococcal vaccine–naïve, community-dwelling adults aged ≥65 years (CAPiTA trial) reported a 75% (95% CI = 41%–91%) vaccine efficacy against VT-IPD and 45% (95% CI = 14%–65%) vaccine efficacy against the first episode of VT-NBPP and noninvasive pneumococcal pneumonia (*3*). This trial excluded adults with immunocompromising conditions upon enrollment. VE estimates from three observational studies ranged from 47% to 68% against VT-IPD in adults aged ≥65 years (*147*–*149*).

Three test-negative, case-control observational studies evaluated PCV13 effectiveness against VT-NBPP with estimates ranging from 38% to 68%. Each study noted that most participants had ≥1 underlying or high-risk condition (*128*,*150*,*151*). A population-based cohort study of approximately 2 million adults aged ≥50 years in Spain did not demonstrate a protective effect of PCV13 against pneumococcal pneumonia or all-cause pneumonia, although the characteristics of those who received PCV13 were significantly different from those who did not receive PCV13, including older age and a higher proportion of adults with immunocompromising conditions among the PCV13 recipients compared with nonrecipients (*152*). Other observational studies have demonstrated the effectiveness of PCV13 against all-cause pneumonia (range = 5%–12%) (*153*–*156*).

Among adults without immunocompromising chronic medical conditions (i.e., adults with chronic heart, lung, or liver disease; asthma; or diabetes mellitus), post hoc analysis of the CAPiTA trial observed a 77% (95% CI = 44%–91%) vaccine efficacy against VT-IPD (*157*) and 40% (95% CI = 11%–60%) vaccine efficacy against a first episode of VT-pneumococcal pneumonia (*158*). Efficacy and effectiveness data regarding PCV13 in immunocompromised adults are limited. A test-negative study of Italian adults aged ≥65 years with immunocompromising or chronic medical conditions observed a 40% (95% CI = −128% to 89%) VE against VT-CAP (*151*).

#### Duration of Protection

Post hoc time-to-event analyses of the CAPiTA trial found a significant difference in disease-free survival among PCV13 recipients compared with placebo recipients for all outcomes throughout the duration of the trial (mean follow-up approximately 4 years). Vaccine efficacy against VT-NBPP and noninvasive pneumococcal pneumonia was 44% 1 year after vaccination and 45% ≤5 years after vaccination; vaccine efficacy against VT-IPD was 67% 1 year after vaccination and 75% ≤5 years after vaccination (*159*). An additional post hoc analysis of data from the CAPiTA trial among adults with chronic medical conditions (excluding those with immunocompromising conditions) also demonstrated significant and persistent efficacy of PCV13 against VT-CAP for an average duration of 4 years (*158*).

#### Safety

A postlicensure review of the safety of PCV13 was conducted using VAERS data during June 2012–December 2015 among adults aged ≥19 years (*160*). VAERS received 2,976 PCV13 reports; 2,800 (94.1%) were nonserious, 2,511 (86%) were in persons aged ≥65 years, and 465 (14%) were in persons aged 19–64 years. Injection site erythema (28%), injection site pain (24%), and fever (22%) were the most frequent adverse events (on the basis of MedDRA preferred terms) among persons aged 19–64 years. The most common adverse events in persons aged ≥65 years were injection site erythema (30%), erythema (20%), and injection site swelling (18%) among those who were administered PCV13 alone. The results of this study were consistent with safety data from prelicensure studies of PCV13.

A more recent review of the safety of PCV13 using VAERS data during January 2016–August 2022 found 17,033 reports (CDC, unpublished data, September 2022). The most common adverse events were local reactions similar to the previous publication (*160*). Data mining analysis did not reveal disproportionate reporting of any new or unexpected adverse events.

A cohort study at six sites from the VSD cohort during January 1, 2011–August 15, 2015, assessed the safety of PCV13 in adults aged ≥65 years compared with PPSV23 for increased risk for certain prespecified adverse events (i.e., cardiovascular events, Bell’s palsy, Guillain-Barré syndrome, syncope, erythema multiforme, thrombocytopenia, cellulitis and infection, allergic reaction, and anaphylaxis). A total of 313,136 doses administered of PCV13 and 232,591 doses of PPSV23 were included. No increased rate of adverse events after PCV13 administration in persons aged ≥65 years compared with PPSV23 was observed (*161*).

### 15-Valent Pneumococcal Conjugate Vaccine (PCV15)

PCV15 contains pneumococcal polysaccharide serotypes 22F and 33F in addition to the PCV13 serotypes, conjugated to CRM197 (Figure). Each 0.5-mL dose of vaccine contains 2.0 *μ*g each of *S. pneumoniae* polysaccharide serotypes 1, 3, 4, 5, 6A, 7F, 9V, 14, 18C, 19A, 19F, 22F, 23F, and 33F and 4.0 *μ*g of 6B, 30 *μ*g of CRM197 carrier protein, 1.55 mg of L-histidine, 1 mg of polysorbate 20, 4.5 mg of sodium chloride, and 125 *μ*g of aluminum as aluminum phosphate adjuvant (*116*).

#### Immunogenicity in Adults Without Previous Pneumococcal Vaccination

Phase II and III RCTs evaluated the immunogenicity and safety of 1 dose of PCV15 compared with PCV13 in multiple populations: healthy adults aged ≥50 years (*162*,*163*), adults aged 18–49 years who are American Indian or Alaska Native (a population with higher rates of IPD than the general U.S. population) (*164*), adults with ≥1 risk condition for pneumococcal disease (*165*), and adults aged ≥18 years with HIV infection (CD4 cell count of ≥50 cells/*μ*L and plasma HIV RNA of <50,000 copies/mL) (*166*). Serotype-specific IgG GMCs and functional antibody responses using an opsonophagocytic activity (OPA) assay were measured 1 month after vaccination. Correlates of protection against pneumococcal diseases have not been established for adults. In one phase II RCT, adults aged ≥50 years were randomized to receive either PCV13, PCV15, or PPSV23. Primary and secondary endpoints were assessment of noninferiority^†^ of PCV15 compared with PPSV23, which were met for all 14 shared serotypes; IgG GMC and OPA geometric mean antibody titers (GMTs) were numerically higher for 13 of 14 serotypes (except for serotype 19F). Compared with PCV13, PCV15 induced numerically higher IgG GMCs and OPA GMTs for eight of 13 and seven of 13 shared serotypes, respectively; noninferiority was not assessed for these comparisons (*162*). In one phase III RCT among adults aged ≥50 years, noninferiority criteria^§^ were met for PCV15 compared with PCV13 for the 13 shared serotypes, and OPA responses were statistically higher^¶^ for shared serotype 3 and PCV15-unique serotypes 22F and 33F (*163*). In studies that evaluated the immunogenicity of PCV15 or PCV13 followed by PPSV23 2–12 months later (*165*–*167*), persons who received PCV15 had numerically similar or higher OPA GMTs for 9–13** shared PCV13 serotypes and a higher percentage of seroresponders (defined as subjects with a greater than fourfold rise in OPA GMT titer postvaccination compared with pre-PCV vaccination) for 5–11 shared serotypes compared with persons who received PCV13 when measured 1 month after receipt of PPSV23. Thus, immunogenicity of PCV13 followed by PPSV23 was considered to be generally comparable to immunogenicity of PCV15 followed by PPV23.

#### Immunogenicity in Adults With Previous Pneumococcal Vaccination

One phase II trial assessed the safety and immunogenicity of a dose of PCV15 compared with PCV13 among adults aged ≥65 years who previously received PPSV23 ≥1 year before PCV receipt (*168*). Compared with baseline, increases of IgG GMCs and OPA GMTs were observed for all PCV serotypes when measured 1 month after PCV administration. Compared with those who received PCV13, those who received PCV15 had numerically similar or higher IgG GMCs and OPA GMTs for the 13 serotypes contained in PCV13 and higher immune responses for PCV15-unique serotypes 22F and 33F when measured 1 month after PCV administration.

#### Safety

Safety of PCV15 was assessed in seven RCTs with 5,630 participants aged ≥18 years who received a single dose of PCV15. Most participants were immunocompetent; however, one study included 302 adults with HIV infection (*166*). Participants from these seven RCTs included persons who had been vaccinated with PPSV23 ≥1 year before receiving PCV15, those who received PCV15 followed by PPSV23, and those who received PCV15 concomitantly with a seasonal inactivated quadrivalent influenza vaccine (IIV4). The most frequently reported adverse reactions were injection site pain, fatigue, and myalgia. The rates of SAEs within 6 months of vaccination in a subset of studies (n = 4,751 for PCV15; n = 1,532 for PCV13) that assessed this outcome were 2.5% among PCV15 recipients and 2.4% among PCV13 recipients. No SAEs or deaths were considered to be related to the study vaccines (*116*,*169*). Postlicensure safety data are being collected and reviewed; however, limited data are available.

### 20-Valent Pneumococcal Conjugate Vaccine (PCV20)

PCV20 contains pneumococcal polysaccharide serotypes 8, 10A, 11A, 12F, 15B, 22F, and 33F in addition to PCV13 serotypes, conjugated to CRM197 (Figure). Each dose of vaccine contains approximately 2.2 *μ*g of all serotypes except for 4.4 *μ*g of 6B saccharides, 51 *μ*g of CRM197 carrier protein, 100 *μ*g of polysorbate 80, 295 *μ*g of succinate buffer, 4.4 mg of sodium chloride, and 125 *μ*g of aluminum as aluminum phosphate adjuvant (*117*).

#### Immunogenicity Among Adults Without Previous Pneumococcal Vaccination

A phase II study among adults aged 60–64 years and two phase III RCTs among adults aged ≥18 years evaluated immunogenicity and safety of PCV20 compared with PCV13 (*170*–*172*). Immunogenicity of PCV20 for the seven additional serotypes included in PCV20 but not in PCV13 was compared with PPSV23 (administered 1 month after PCV13) (*170*,*172*). These studies included adults with stable medical conditions (i.e., disease not requiring significant change in therapy or hospitalization for worsening disease during the weeks preceding receipt of the vaccine) but not adults with immunocompromising conditions. Compared with PCV13 recipients, PCV20 recipients elicited responses that met the noninferiority criterion (defined as the lower bound of the two-sided 95% CI of the ratio [PCV20/PCV13] of OPA GMT being >0.5) for all 13 serotypes in a phase III trial among adults aged ≥60 years (*170*); however, PCV20 recipients appeared to have lower GMTs and included a lower percentage of seroresponders to 12–13 of the 13 PCV13-shared serotypes (*170*,*172*). The clinical relevance of these findings is unknown. Compared with PPSV23 recipients, PCV20 recipients had numerically higher GMTs and a higher percentage of seroresponders to six of seven (excluding serotype 8) shared non-PCV13 serotypes (*170*,*172*); the noninferiority criterion was met for those six serotypes (*170*).

#### Immunogenicity Among Adults With Previous Pneumococcal Vaccination

Two phase III studies assessed the immunogenicity of PCV20 among adults aged ≥65 years who had previous pneumococcal vaccination (PCV13 only, PPSV23 only, or both PCV13 and PPSV23) (*173*,*174*). These studies included adults with stable medical conditions, but not adults with immunocompromising conditions. Immunogenicity to PCV20 was assessed 1 month after receipt of PCV20. In one study, participants either received PCV13 ≥6 months previously; PPSV23 1–5 years previously; or PCV13 followed by PPSV23, with PPSV23 ≥1 year previously (*173*). The other study was a post hoc analysis of a subset of participants who received pneumococcal vaccines (PCV13, PPSV23, or both) ≥6 months previously in a phase III clinical trial of the coadministration of PCV20 with a seasonal inactivated influenza vaccine (*174*,*175*). Neither study directly compared the immunogenicity of PCV20 with PPSV23 after receipt of both PCV13 and PPSV23. Compared with persons who previously received PPSV23 only, those who previously received both PCV13 and PPSV23 had numerically higher OPA GMTs of serotypes included in PCV20 in 19 of 20 PCV20 serotypes in one study (*173*) and for 15 of 20 PCV20 serotypes in another study (*174*). In addition, those who received PCV13 only had numerically higher OPA GMTs of serotypes included in PCV20 compared with adults who previously received PPSV23. Percent seroresponders (defined as a greater than fourfold rise in OPA titer from before to 1 month after PCV20 receipt) was numerically higher for 13–18 of 20 serotypes among adults who previously received PCV13 compared with adults who previously received PPSV23. Compared with adults who previously received PPSV23 only, percent seroresponders were numerically higher for four of 20 serotypes for adults who received both PCV13 and PPSV23 in one study (*173*) and for six of 20 serotypes in another study (*174*,*175*). Percent seroresponders in these studies typically were lower compared with those observed among pneumococcal vaccine–naïve adults (*170*). Regardless, these data demonstrate that PCV20 is immunogenic in those aged ≥65 years who previously received a pneumococcal vaccine.

#### Safety

##### Clinical Trials

Safety was assessed in seven trials among immunocompetent adults aged ≥18 years; the trials included a total of 6,343 participants who received PCV20 (*117*). Participants included persons who were naïve to pneumococcal vaccination and those who had previously received pneumococcal vaccination. In the pivotal phase III trial among pneumococcal vaccine–naïve adults, the most frequently reported adverse reactions were injection site pain, muscle pain, fatigue, headache, and joint pain (55%, 39%, 30%, 22%, and 13%, respectively, among adults aged ≥60 years; 50%–61%, 50%–67%, 40%–43%, 32%–39%, and 13%–15%, respectively, among adults aged 18–59 years) (*117*). Among participants across six of seven trials, SAEs reported within 6 months after vaccination occurred among 1.5% of PCV20 recipients (n = 4,552) and among 1.8% of controls (n = 2,496) (*117*). No SAEs or deaths were considered to be related to study vaccines across all seven trials (*117*,*176*).

##### Postlicensure Safety Monitoring

A postlicensure review of the safety of PCV20 was conducted using VAERS data during October 21, 2021–August 31, 2022. A total of 412 reports related to PCV20 were submitted to VAERS; 399 (96.8%) were nonserious, 389 (94.4%) were in adults aged ≥19 years, and 313 (75.9%) were in females. Thirteen SAEs were reported, including two deaths. Cause of death in a report of a female aged 51 years was acute brain herniation secondary to acute left temporal hemorrhage; cause of death in the second report was not specified. Among the 11 remaining SAEs reported, the most common adverse events were asthenia, nausea, fever, and gait instability. When PCV20 was administered alone (n = 274) the most common adverse events were injection site erythema (26.6%), injection site swelling (22.6%), and injection site pain (22.3%). These proportions are similar to commonly reported adverse events across all vaccines approved for use in adults: injection site erythema (22.6%), injection site pain (19.3%), and erythema (19%). Data mining analysis did not reveal disproportional reporting of any new or unexpected adverse events after PCV20 administration.

### Immune Response After Repeat PCV Dosing

#### Adults Without Immunocompromising Conditions

Findings from six immunogenicity studies that evaluated responses after repeat PCV administration were reviewed. In two studies among pneumococcal vaccine–naïve adults aged ≥60 years, immune responses after a second PCV13 dose, 1 year after the initial PCV13 dose, were statistically significantly lower for the majority of serotypes compared with the initial PCV13 dose (*177*,*178*). In contrast, in two studies among pneumococcal vaccine–naïve adults aged ≥50 years, immune responses after a second PCV13 dose, delivered 3.5–5 years after the initial PCV13 dose, were comparable or statistically significantly greater for the majority of serotypes compared with the initial PCV13 dose (*179*,*180*). These findings suggest that longer intervals between PCV13 administrations might optimize immune responses to subsequent doses. In another study among adults aged ≥70 years who had received 1 dose of PPSV23 ≥5 years before enrollment, two administrations of PCV13, 1 year apart, resulted in a recovery of the responses to levels similar to those after the initial PCV13 dose, indicating that an initial dose of PCV13 does not have a negative impact on immune responses to subsequent PCV13 doses among adults previously vaccinated with PPSV23 adults (*181*). Finally, in post hoc analysis among adults who had received PCV13 ≥6 months before, immune responses 1 month after administration of PCV20 compared with baseline (i.e., day of PCV20 vaccination) were numerically higher for the 13 serotypes common to PCV13 and PCV20 as well as for the seven additional serotypes included in PCV20 (*182*). The percentage of subjects with a greater than fourfold rise in OPA titers was typically lower in this study compared with the percentage observed in the pivotal trial among pneumococcal vaccine–naïve adults (*170*).

#### Adults With Immunocompromising Conditions

In one study among adults aged ≥18 years with HIV infection who previously received PPSV23 ≥6 months before the first PCV13 dose, IgG GMCs to the PCV13 serotypes were measured 1 month after the second and third doses of PCV13 that were administered 6 months apart. IgG GMCs after the second and third doses of PCV13 were comparable to those after the first dose of PCV13 (*183*). In another study among pneumococcal vaccine–naïve persons aged ≥6 years with HIV infection who received 3 doses of PCV13 followed by a single dose of PPSV23 1 month apart, the largest increase in IgG GMCs or OPA GMTs for the PCV13 serotypes was after the first dose, and only modest increases were observed after the second and third doses (*184*).

### Adults Who Received HSCT

HSCT recipients can have as high as 80 times the risk for IPD compared with the general population (*86*). HCST recipients have a poor response to PPSV23 when administered during the first year of transplantation or later, especially those with chronic graft versus host disease (GVHD) (*185*,*186*). Because of the prolonged process of lymphocyte recovery after HSCT, both children and adults who are HSCT recipients have been recommended to receive 3 doses of PCV13 starting at 3–6 months after HSCT (*15*,*92*). PPSV23 as the fourth dose of pneumococcal vaccine starting 12 months after HSCT has been found to induce immune responses to vaccine serotypes (*187*) and has been recommended to provide immunity to additional serotypes that are not included in PCVs, except for patients with chronic GVHD who have been recommended to receive PCV for their fourth dose because they are unlikely to respond to PPSV23 (*92*).

In a phase III RCT, children and adults who received allogenic HSCT were randomized to receive either 3 doses of PCV13 or PCV15 starting 3–6 months after HSCT, followed by a single dose of PPSV23 at 12 months (or a single dose of PCV13 or PCV15 for those who developed GVHD) (*188*). Immunogenicity measured 1 month after the third PCV dose indicated that IgG GMC and OPA GMT were typically comparable for the 13 shared PCV13 serotypes, and PCV15 had significantly higher immunogenicity for two additional serotypes not included in PCV13. In both intervention groups, PPV23 also was immunogenic (as assessed by geometric mean fold rises [GMFRs] in IgG GMC and OPA GMT) from prevaccination (month 12) to 30 days postvaccination with PPV23 (month 13) for the 13 shared serotypes in PCV13 and for the two additional serotypes unique to PCV15 (*189*,*190*). Serotype-specific IgG GMCs and OPA GMTs measured in month 13 were typically comparable between the two groups for the 13 shared serotypes contained in PCV13; compared with PCV13 recipients, PCV15 recipients had numerically higher IgG GMC and OPA GMT values for the two serotypes unique to PCV15. A fourth dose of PCV15 in patients with chronic GVHD also was immunogenic for all 15 serotypes contained in the vaccine (as assessed by GMFRs) from prevaccination (month 12) to 30 days postvaccination. The frequency of vaccine-related adverse events was higher in HSCT recipients aged ≥18 years who received PCV15 compared with those who received PCV13 after receiving 3 doses of PCV (injection-site adverse events: 93.1% versus 75.2%; systemic adverse events: 55% versus 43.4%) although the majority of adverse events were mild to moderate. The frequency of adverse events after receipt of PPSV23 at 12 months (injection-site adverse events: 71.4% versus 64.8%; systemic adverse events: 31.0% versus 29.6%) were comparable between the PCV15 and PCV13 groups. No clinical trials of PCV20 among HSCT recipients have been reported.

The optimal timing of vaccination among HSCT recipients has been discussed globally. The Dutch immunization schedule recommends starting vaccination 1 year after HSCT to allow time for immune reconstitution after allogenic HSCT (*191*). Alternatively, starting vaccination earlier gives the opportunity for HSCT recipients to have protection against pneumococcal disease earlier (*186*). In a trial that compared initiating 3 doses of PCV7 3 months or 9 months after HSCT followed by a dose of PPSV23 12 or 18 months after HSCT, the response rate (defined as the percentage of patients with antibody titers of ≥0.15 *μ*g/mL to all the PCV7 serotypes 1 month after the third PCV7 dose) was not statistically significantly different between the two groups, although the GMC and response rates were lower in the early vaccination group at 24 months (*192*). For the serotypes unique to PPSV23, the early vaccination group had lower GMCs and percentage of responders at 24 months, although the differences between the two groups were not statistically significant (*187*).

To provide earlier and prolonged protection against IPD after HSCT, use of 4 doses of PCV13 (3 doses administered 1 month apart followed by a booster dose 6 months after the third dose of PCV) followed by a single dose of PPSV23 has been studied (*193*,*194*). One study assessed immunogenicity and safety of 4 doses of PCV13 doses followed by a single dose of PPSV23 1 month later among children and adults who received HSCT 3–6 months previous. Results indicated that the GMFR of IgG concentrations from baseline to 1 month after the third dose and from after the third dose to 1 month after the fourth dose increased significantly across all PCV13 serotypes in both children and adults; however, there was little change in the GMFR when comparing 1 month after the fourth dose with 1 month after the PPSV23 dose (*194*). Similar findings were reported in a separate study using a similar 5-dose vaccine schedule (4 doses of PCV13 followed by 1 dose of PPSV23 2 months after the fourth dose of PCV13) among adults who received allogenic HSCT 3–6 months previous (*193*). Of note, the interval between PCV and PPSV23 doses used in these trials was shorter than the current recommended schedule. Although these studies do not provide direct comparison of a schedule consisting of 4 doses of PCV with a schedule using 3 doses of PCV13 and 1 dose of PPSV23 at 12 months, findings from these studies suggest that use of 4 doses of PCV is immunogenic and the booster dose might provide additional protection against the serotypes covered in the vaccine. Regarding safety of using 4 doses of PCV among HSCT recipients, local and systemic reactions occurred more frequently after the fourth dose of PCV13, compared with after the first, second, and third doses of PCV13 (*194*). Vaccine-related SAEs occurred across vaccine doses in one study, including facial diplegia, injection site erythema and pyrexia, autoimmune hemolytic anemia, Guillain-Barré syndrome, and cellulitis (*194*).

### Intervals Between PCV and PPSV23

Seven immunogenicity studies evaluating the immune response after a sequence of PCV13 or PCV15 followed by PPSV23 administered at intervals of 2, 6, or 12 months or 3–4 years were reviewed (*165*–*167*,*177*,*179*,*195*,*196*). Four were among adults aged ≥50 years without immunocompromising conditions (*167*,*177*,*179*,*195*). Three included younger adults (aged ≥18 years) who are considered to be at increased risk for pneumococcal disease: one among American Indian and non-American Indian adults with chronic medical conditions (*165*), one among adults with HIV infection (*166*), and one among adults with rheumatoid arthritis receiving biologic disease-modifying antirheumatic drugs (*196*). In addition to these seven studies, one PCV7 study that compared different intervals between PCV7 and PPSV23 among Alaska Native adults aged 55–70 years was included (*197*). Of eight studies, three that compared intervals ranging from 2 to 6 months between administration of PCV and PPSV23 in the same study found no significant difference in immunogenicity measured after PPSV23 receipt, although reactogenicity tended to be higher with shorter intervals (*195*–*197*). In a study conducted among pneumococcal vaccine–naïve adults aged 60–64 years that compared antibody responses to 1 dose of PCV13 with responses to PCV13 followed by PPSV23 1 year apart, the immune responses after PPSV23 were significantly lower compared with the responses after 1 dose of PCV13 for eight of 12 common serotypes (*177*). In another study among pneumococcal vaccine–naïve adults aged ≥50 years who received either PCV13 or PCV15 followed by PPSV23 1 year apart, immune responses (measured as GMFR in IgG concentrations and a greater than fourfold rise in OPA titers 1 month after vaccination compared with prevaccination) to PPSV23 were typically comparable to those measured 1 month after 1 dose of PCV for the serotypes contained in the PCVs (*167*). In another study that was conducted as an extension of a previous study in pneumococcal vaccine–naïve adults aged 50–64 years, antibody response to 1 dose of PCV13 was compared with responses to PCV13 followed by PPSV23 approximately 3.5 years apart (*179*). Immune responses after PPSV23 were significantly higher for seven of 12 common serotypes (*179*). These findings suggested that longer intervals between administration of PCV and PPSV23 might improve immunogenicity in immunocompetent adults, although a direct comparison between a 1- versus 4-year interval was not made.

### Coadministration With Other Vaccines

Concomitant administration of PCV15, PCV20, or PPSV23 with seasonal inactivated quadrivalent influenza vaccine (specifically, PCV15 or PPSV23 and IIV4 [Fluarix] and PCV20 and adjuvanted IIV4 [Fluad]) has been demonstrated to be immunogenic and safe in adults (*174*,*198*,*199*). However, numerically lower pneumococcal serotype–specific OPA GMTs or GMCs were reported for certain serotypes when pneumococcal vaccines were coadministered with IIV4 compared with administration of pneumococcal vaccines alone.

Among adults aged ≥65 years who received 2 doses of BNT162b2 COVID-19 vaccine with the second BNT162b2 dose administered ≥6 months previous, slightly lower pneumococcal serotype–specific OPA GMTs were reported when PCV20 was coadministered with BNT162b2 compared with administration of PCV20 alone; however, this difference was not statistically significant. Pain at injection site within 10 days after vaccination and systemic events within 7 days after vaccination were reported more frequently among the group that received both PCV20 and BNT162b2 compared with the group that received PCV20 alone. The percentage of participants who reported any adverse events through 1 month after vaccination was similar across groups (PCV20 and BNT162b2 coadministration: 5.3% [95% CI = 2.6%–9.6%]; PCV20 only: 4.3% [95% CI = 1.9%–8.3%]; BNT162b2 only: 6.5% [95% CI = 3.4%–11.1%]) (*200*). No data are available on PCV20 coadministration with other COVID-19 vaccines. Evaluation of coadministration of PCV15 or PPSV23 with COVID-19 vaccines among adults aged ≥50 years is ongoing (*201*). In adults aged ≥50 years who received 2 doses of adjuvanted recombinant zoster vaccine (RZV), immune responses to coadministration of RZV and PPSV23 were noninferior to those in this age group who received these vaccines in sequence. Solicited adverse reactions were reported more frequently when the first doses of RZV and PPSV23 were coadministered (*202*). No data are available on coadministration with other vaccines (e.g., tetanus, diphtheria, acellular pertussis vaccine, or hepatitis B vaccine) in adults.

## Economic Assessment of Use of PCVs

Available economic evidence on the use of pneumococcal conjugate vaccines in the United States primarily comes from three cost-effectiveness models from Tulane-CDC, Merck, and Pfizer. These models summarize the economic value of pneumococcal conjugate vaccines by calculating a cost-effectiveness ratio in terms of cost per quality-adjusted life-year (QALY) gained. A QALY is a measure of health-related quality of life that uses numeric values to represent how disease episodes can affect the morbidity and mortality of a population at risk for disease. In an economic model that uses QALYs, in a given year, the QALY of a person can range from 0.0 to 1.0, where a value of 1.0 indicates a year of perfect health, a value of <1.0 but >0.0 represents a year of imperfect health, and a value of 0.0 indicates death. All cost-effectiveness ratios included in this report have been adjusted to 2022 U.S. dollars using the consumer price index. As a consequence, numeric values in this report might not exactly match the values reported previously.

### Use of PCV15 or PCV20 in Adults Who Previously Have Not Been Vaccinated

When PCV15 and PCV20 recommendations were made for adults who have previously not been vaccinated, three economic models were reviewed, one each from Tulane-CDC, Merck, and Pfizer (*16*,*203*) (Table 8). These economic models assessed cost-effectiveness of policy options on the use of PCV15 or PCV20 compared with previous recommendations (PPSV23 only for adults aged 19–64 years with chronic medical conditions; PPSV23 with an option to receive PCV13 on the basis of shared clinical decision-making for adults aged ≥65 years without an immunocompromising condition, a CSF leak, or a cochlear implant; and both PCV13 and PPSV23 for adults with an immunocompromising condition, a CSF leak, or a cochlear implant) (*14*). The Tulane-CDC, Merck, and Pfizer economic models assessed PCV20 alone for all adults aged ≥65 years with cost-effectiveness estimates ranging from cost saving (i.e., lower cost and improved health outcomes compared with previous recommendations) to $42,000 per QALY gained (*16*,*203*). Two economic models (Tulane-CDC and Merck) assessed use of PCV15 in series with PPSV23 for all adults aged ≥65 years with estimates ranging from cost saving to $309,000 per QALY gained. The Tulane-CDC model found cost savings in all scenarios for use of either PCV20 alone or PCV15 in series with PPSV23 for all adults aged ≥65 years. Cost-effectiveness estimates of policy options for adults aged 19–64 years with certain underlying medical conditions or other risk factors ranged from $13,000 to $320,000 per QALY gained for use of PCV20 alone and from $274,000 to $718,000 for use of PCV15 in series with PPSV23 (Table 8).

**TABLE 8:** Selected characteristics and results of cost-effectiveness analysis models assessing PCV15 or PCV20 use among previously unvaccinated adults, adjusted to 2022 U.S. dollars

Characteristic	Tulane-CDC	Merck	Pfizer
Model type	Single cohort, lifetime	Multicohort, lifetime	Multicohort, lifetime
One base case or scenarios	One base case	Four scenarios	One base case
Societal perspective or health care sector	Societal	Societal	Both
Adverse events	No	No	No
Combined age- and risk-based estimates	Yes	No	Yes
Transitions from lower risk to higher risk (e.g., healthy to chronic medical conditions)	Yes	No	Yes
Indirect effects from potential pediatric vaccinations with new vaccines	In scenarios	Not included	In base case
Lower PCV VE against serotype 3 disease compared with other PCV serotypes	In base case	In base case scenarios	In scenarios
PPSV VE against NBPP >0%	In base case	In base case scenarios	No
Vaccination coverage in the intervention vs. current recommendations	Varies in scenarios	PCV coverage higher	PCV coverage higher
Comparator in the risk-based analysis	Age-based use (incremental)	Status quo*	Status quo*
Important inputs and assumptions on basis of available sensitivity analyses	Indirect effects; VE (initial, waning)	VE (initial, waning)	Indirect effects VE (initial, waning) NBPP incidence
PCV20 use for all adults aged ≥65 yrs vs. current recommendations	Cost saving^†^	Cost saving^†^ to $42,000^§^ per QALY gained	Cost saving
PCV15 use in series with PPSV23 for all adults aged ≥65 yrs vs. current recommendations	Cost saving^†^	$260,000–$309,000^§^ per QALY gained; cost saving^†^ in sensitivity analysis	Not assessed
Risk-based PCV20 use for adults aged 19–64 yrs	$320,000^§^ per QALY gained	$63,000–$201,000^§^ per QALY gained	$13,000^¶^ per QALY gained
Risk-based PCV15 use in series with PPSV23 for adults aged 19–64 yrs	$718,000^§^ per QALY gained	$274,000–$342,000^§^ per QALY gained	Not assessed

**Abbreviations:** NBPP = nonbacteremic pneumococcal pneumonia; PCV = pneumococcal conjugate vaccine; PCV13 = 13-valent pneumococcal conjugate vaccine; PCV15 = 15-valent pneumococcal conjugate vaccine; PCV20 = 20-valent pneumococcal conjugate vaccine; PPSV23 = 23-valent pneumococcal polysaccharide vaccine; QALY = quality-adjusted life-year; USD = U.S. dollars; VE = vaccine effectiveness.

* **Source:** Matanock A, Lee G, Gierke R, Kobayashi M, Leidner A, Pilishvili T. Use of 13-valent pneumococcal conjugate vaccine and 23-valent pneumococcal polysaccharide vaccine among adults aged ≥65 years: updated recommendations of the Advisory Committee on Immunization Practices. MMWR Wkly Rep 2019;68:1069–75.

^†^ Lower cost and improved health outcomes compared with previous recommendations.

^§^ Original report was in 2021 USD, multiplied by 1.10 to adjust to 2022 USD.

^¶^ Original report was in 2020 USD, multiplied by 1.15 to adjust to 2022 USD.

Other cost-effectiveness estimates evaluating PCV20 and PCV15 use have been published (*204*,*205*). In one study that compared higher-valency vaccines used for all adults aged ≥65 years with recommendations in place in early 2021 (i.e., routine use of PPSV23 and PCV13 on the basis of shared clinical decision-making), cost-effectiveness estimates ranged from $209,000 to $544,000 per QALY gained for use of PCV20 alone and from $531,000 to $676,000^††^ per QALY gained for use of PCV15 in series with PPSV23 with the ranges resulting from the inclusion of indirect effects (*205*). A follow-up analysis by the same research group largely confirmed the initial findings; in particular, use of PCV15 in series with PPSV23 had higher estimates of cost per QALY gained than use of PCV20 alone (*204*).

### Use of PCV20 in Adults Who Previously Received PCV13

#### Adults Who Received PCV13 Only

When PCV20 was recommended for adults who previously received PCV13 only, the Tulane-CDC, Merck, and Pfizer models were reviewed (*175*). In the Tulane-CDC model, the cost per QALY gained of PCV20 use 1 or 5 years after receipt of PCV13 compared with no vaccine in a cohort of adults aged ≥65 years was $540,000, with a fifth and 95th percentile range of $438,000–$812,000 in probabilistic sensitivity analyses. In a cohort of adults aged 42 years (age selected to represent adults aged 19–64 years) with immunocompromising conditions, the cost per QALY gained of PCV20 use 1 or 5 years after receipt of PCV13 compared with no vaccine use was $279,000 with a fifth and 95th percentile range of $197,000–$540,000 in probabilistic sensitivity analyses. The Tulane-CDC model included multiple scenarios that varied age of vaccination with PCV20, time since previous vaccination, indirect effects, and PCV VE against serotype 3 disease. Across these scenarios in the Tulane-CDC model, the cost per QALY gained varied from $209,000 to $403,000.

Because of the small estimated population size of adults aged 19–64 years with immunocompromising conditions who have received PCV13 only (*206*,*207*), a comparison across the three models focused on adults aged ≥65 years who have received PCV13 only compared with no vaccine (Table 9). Across the three models, cost per QALY gained of PCV20 use ranged from $87,000 (Pfizer model: PCV20 administered 7 years after the PCV13 dose) to $611,000 (Tulane-CDC model: PCV20 administered to adults aged 66 years with an immunocompromising condition 1 year after the PCV13 dose).

**TABLE 9:** Selected characteristics and results of cost-effectiveness analysis models assessing 20-valent pneumococcal conjugate vaccine use among adults aged ≥65 years who previously received 13-valent pneumococcal conjugate vaccine, adjusted to 2022 U.S. dollars

Characteristic	Tulane-CDC	Merck	Pfizer
Model type	Single cohort,* lifetime	Single cohort,* lifetime	Multicohort,^†^ lifetime
Perspective	Societal^§^	Health care^§^	Health care
Adverse events	No	No	No
Sensitivity analyses	Univariate, scenario, and probabilistic sensitivity analyses	Univariate, scenario, and probabilistic sensitivity analyses	Univariate and scenario analyses
Indirect effects from pediatric vaccination	Yes^¶^	No	Yes^¶^
Lower PCV VE against serotype 3 disease compared with other PCV serotypes	In base case	In base case scenarios	In scenarios
PPSV23 VE against NBPP	7%–20%**	3%–67%**	0%
Inpatient NBPP case-fatality ratios	3%–5%^††^	7%–12%^††^	3%–11%^††^
QALY loss for IPD and inpatient NBPP	0.071	0.071	0.130
**PCV20 use for adults aged ≥65 yrs who received PCV13 only vs. no additional vaccine**			
Time since previous vaccination	1 yr	5 yrs	7 yrs
Age at PCV20 vaccination, yrs	66 and 76	73	65–99 (average 75)
Cost per QALY gained	$139,000–$611,000^§§^	$182,000^¶¶^	$87,000***
**PCV20 use for adults aged ≥65 yrs who received both PCV13 and PPSV23 vs. no additional vaccine**			
Time since previous vaccination	1, 5, and 10 yrs	5 yrs	7 yrs
Age at PCV20 vaccination, yrs	67, 71, 76, 77, and 81 (without immunocompromising conditions); 66, 70, 75, 76, and 80 (with immunocompromising conditions)	73	65–99 (average 75)
Cost per QALY gained	$168,000–$970,000** (without immunocompromising conditions); $421,000–$925,000** (with immunocompromising conditions)	$217,000	$93,000–$182,000^¶¶^

**Abbreviations:** IPD = invasive pneumococcal disease; NBPP = nonbacteremic pneumococcal pneumonia; PCV = pneumococcal conjugate vaccine; PCV13 = 13-valent pneumococcal conjugate vaccine; PCV15 = 15-valent pneumococcal conjugate vaccine; PCV20 = 20-valent pneumococcal conjugate vaccine; PPSV23 = 23-valent pneumococcal polysaccharide vaccine; QALY = quality-adjusted life-year; USD = U.S. dollars; VE = vaccine effectiveness.

* In any scenario, the Tulane-CDC and Merck models start with a single-aged cohort of individuals, not a full population composed of many different ages. For this reason, these models have one specific starting age. For example, a cohort aged 70 years might be used to represent and estimate values in the group aged ≥65 years.

^†^ The Pfizer model base case used a multicohort model; however, single cohort results were provided in a scenario.

^§^ The Tulane-CDC model includes one societal perspective component (i.e., travel cost added to the cost of vaccine administration). The Merck model provided a scenario using the societal perspective that was similar to the Tulane-CDC model.

^¶^ The Tulane-CDC and Pfizer models included scenarios where no herd effects from pediatric vaccinations occurred.

** The Tulane-CDC model varied PPSV23 VE against NBPP across two risk groups (adults with or without immunocompromising conditions). The Merck model varied PPSV23 VE against NBPP across three risk groups (low risk, at risk, and high risk).

^††^ The Tulane-CDC and Merck models varied inpatient NBPP case-fatality ratios by age. The Pfizer model varied inpatient NBPP case-fatality ratios by age and risk group.

^§§^ Original report was in 2021 USD, multiplied by 1.10 to adjust to 2022 USD.

^¶¶^ The Merck model did not report a value for the comparison. The incremental value was calculated by the CDC team.

*** Original report was in 2020 USD, multiplied by 1.15 to adjust to 2022 USD.

 The Merck model did not report a value for this comparison. However, on the basis of calculations by the CDC review team, the Merck model estimated values that would be between the Pfizer and Tulane-CDC model estimates.

#### Adults Aged 19–64 Years With Immunocompromising Conditions Who Received Both PCV13 and PPSV23

In the Tulane-CDC model, PCV20 use 1 or 5 years after receipt of both PCV13 and PPSV23 was compared with PPSV23 as previously recommended in a single-age cohort of adults aged 42 years (age selected to represent adults aged 19–64 years) with immunocompromising conditions. This cohort was followed through age 64 years. The cost per QALY gained from PCV20 use ranged from $209,000 to $403,000.

In the Pfizer model, a multiage cohort of adults aged 19–64 years with immunocompromising conditions was followed through their lifetime. Cost per QALY gained ranged from $40,000 (PCV20 administered 7 years since the PCV13 dose, assuming no indirect effects from pediatric PCV20 vaccination) to $107,000 per QALY gained (PCV20 administered 7 years since the PCV13 dose, assuming indirect effects from pediatric PCV20 vaccination).

In the Merck model that followed a single-age cohort of adults aged 42 years with immunocompromising conditions through their lifetime, PCV20 use at 5 years after both PCV13 and PPSV23 administration resulted in higher cost and worse health outcomes compared with the current recommendation strategy of completing the pneumococcal vaccination series with PPSV23. Of note, this model assumed the comparator group receives doses of PPSV23 at ages 52 and 65 years per current recommendation, which is not considered in other models because of the longer time horizon. In addition, the incremental change in QALY was small.

#### Adults Aged ≥65 Years Who Received Both PCV13 and PPSV23

In the Tulane-CDC model, the cost per QALY gained of PCV20 use 5 years after receipt of both PCV13 and PPSV23 compared with no additional vaccination in a single-age cohort of adults aged 65 years was $454,000 with a fifth and 95th percentile range of $308,000–$956,000 in probabilistic sensitivity analyses. In scenario analyses that varied age, years after receipt of both PCV13 and PPSV23, indirect effects, and PCV VE against serotype 3 disease (i.e., no versus moderate protection against serotype 3 disease), the cost per QALY gained of PCV20 use ranged from $168,000 to $970,000 among adults without immunocompromising conditions and from $421,000 to $925,000 among adults with immunocompromising conditions (Table 9). The higher cost per QALY gained among adults with immunocompromising conditions is likely because of the assumptions of shorter life expectancy and the lower VE among this population. In the Pfizer model that assumed PCV20 use 7 years after receiving both PCV13 and PPSV23 at age 65 years, the cost per QALY gained of PCV20 use compared with no additional vaccination ranged from $93,000 without an indirect effects assumption to $182,000 with an indirect effects assumption. In the Merck model that assumed PCV20 use 5 years after receiving both PCV13 and PPSV23 at age 68 years, the cost per QALY gained of PCV20 use compared with no additional vaccination was $217,000.

The cost per QALY gained typically was higher if shorter intervals since the last pneumococcal vaccine dose were used. In the Tulane-CDC model, the modeling of 1-, 5-, and 10-year intervals for adults who received both PCV13 and PPSV23 starting at age 65 years or 75 years ranged from $168,000 per QALY gained (PCV20 administered 5 years since PCV13 and PPSV23 administered starting at age 75 years) to $970,000 per QALY gained (PCV20 administered 1 year since PCV13 and PPSV23 administered starting at age 65 years).

## Recommendations for Use of Pneumococcal Vaccines in Adults Aged ≥65 Years

### Adults Who Previously Have Not Received Any Pneumococcal Vaccine

Adults aged ≥65 years who previously have not received a pneumococcal vaccine, or whose previous vaccination history is unknown, should receive a single dose of PCV20 or PCV15. When PCV15 is used, it should be followed at a ≥1 year interval with a single dose of PPSV23 (Tables 2 and 7). The minimum recommended interval for receipt of the PPSV23 dose in adults with an immunocompromising condition, a CSF leak, or a cochlear implant is ≥8 weeks since the PCV15 dose.

#### Rationale

Phase II and III clinical trials demonstrated that the immunogenicity of PCV15 or PCV20 in older adults who previously have not received a pneumococcal vaccine was noninferior to that of PCV13, PPSV23, or both. Safety profiles of PCV15 and PCV20 were comparable to that of PCV13 (*22*,*24*). PCV15 use is recommended in series with PPSV23 to provide protection against broader serotypes. No clinical trial data of PCV20 use among adults with immunocompromising conditions are available. Limited PCV13 effectiveness data from observational studies that included adults with immunocompromising conditions indicate that PCV13 confers protection against VT-IPD (*147*–*149*) or VT-pneumococcal pneumonia (*151*).

Economic models demonstrated that use of PCV20 alone for adults aged ≥65 years compared with previous recommendations had cost-effectiveness estimates ranging from cost saving to $42,000 per QALY gained (*16*,*203*). Two economic models (Tulane-CDC and Merck) assessed use of PCV15 in series with PPSV23 for all adults aged ≥65 years compared with previous recommendations with estimates ranging from cost saving to $309,000 per QALY gained.

### Adults Who Previously Have Received PCV13 Only

Adults aged ≥65 years who previously have received PCV13 only are recommended to complete their pneumococcal vaccine series by receiving either a dose of PCV20 or PPSV23 with an interval of ≥1 year after the PCV13 dose. When PPSV23 is used for adults with an immunocompromising condition, a CSF leak, or a cochlear implant, the minimum recommended interval between PCV13 and PPSV23 is 8 weeks (*11*,*14*) (Table 2).

#### Rationale

Phase III clinical study data among adults aged ≥65 years demonstrated that PCV20 use in adults without immunocompromising conditions who received PCV13 ≥6 months previous was safe and immunogenic (https://www.cdc.gov/vaccines/acip/recs/grade/PCV20-prev-vax-adults-etr.html). A comparison of three economic models that assessed use of PCV20 for adults aged ≥65 years who previously have received PCV13 only indicated that cost per QALY gained of PCV20 use ranged from $87,000 (Pfizer model: PCV20 administered 7 years after the PCV13 dose) to $611,000 (Tulane-CDC model: PCV20 administered to adults aged 66 years with an immunocompromising condition 1 year after the PCV13 dose) (Table 9). PCV13 was recommended previously for all adults with an immunocompromising condition, a CSF leak, or a cochlear implant or on the basis of shared clinical decision-making for adults aged ≥65 years without an immunocompromising condition, a CSF leak, or a cochlear implant (*14*). If PCV13 was administered, these adults were recommended to complete the series with PPSV23. Although PPSV23 provides the broadest serotype coverage among available pneumococcal vaccines, considering the immunologic advantages of PCVs compared with PPSV23 (*113*–*115*), use of a single dose of PCV20 is an option to PPSV23 to complete the pneumococcal vaccine series for adults who have received PCV13 to provide broader pneumococcal serotype coverage for these adults who are at increased risk for pneumococcal disease.

### Adults Who Previously Have Received Both PCV13 and PPSV23 But Have Not Yet Received a Final Dose of PPSV23

Adults aged ≥65 years who have received both PCV13 and PPSV23 according to previous pneumococcal vaccine recommendations (*14*) but have not yet received a final dose of PPSV23 at age ≥65 years are recommended to complete their pneumococcal vaccine series by receiving either a single dose of PCV20 or PPSV23. If PCV20 is selected, it can be administered ≥5 years after the last pneumococcal vaccine dose. If PPSV23 is selected, it can be administered at an interval ≥1 year since the PCV13 dose (or a minimum interval of ≥8 weeks since the PCV13 dose for adults with an immunocompromising condition, a CSF leak, or a cochlear implant) and ≥5 years since the previous PPSV23 dose (*11*) (Table 2).

#### Rationale

Phase III clinical trial data among adults aged ≥65 years without immunocompromising conditions who received both PCV13 and PPSV23 1–5 years previous demonstrated that PCV20 use is safe and immunogenic (https://www.cdc.gov/vaccines/acip/recs/grade/PCV20-prev-vax-adults-19-64-risk-based-etr.html). Economic models that assessed use of PCV20 for adults aged <65 years with immunocompromising conditions who previously have received PCV13 and PPSV23 had variable results, ranging from $40,000 per QALY gained (Pfizer model: PCV20 administered 7 years since the PCV13 dose, assuming no indirect effects from pediatric PCV20 vaccination) to higher cost and worse health outcomes (Merck model: PCV20 use at 5 years after receipt of both PCV13 and PPSV23) because of differences in the model assumptions. Although PPSV23 provides the broadest serotype coverage among available pneumococcal vaccines, considering the immunologic advantages of PCVs compared with PPSV23 (*113*–*115*), use of a single dose of PCV20 is an option to PPSV23 for adults who have received PCV13 to provide broader pneumococcal serotype coverage for these adults who are at increased risk for pneumococcal disease.

### Adults Who Have Completed Their Recommended Vaccine Series With Both PCV13 and PPSV23

Shared clinical decision-making is recommended regarding administration of PCV20 for any adult aged ≥65 years who has completed the recommended vaccine series with both PCV13 and PPSV23 (i.e., PPSV23 was administered at age ≥65 years) but has not received PCV15 or PCV20. If a decision to administer PCV20 is made, a single dose of PCV20 is recommended ≥5 years after the last pneumococcal vaccine dose. Considerations for shared clinical decision-making might include the patient’s risk for pneumococcal disease due to underlying medical conditions, time since last pneumococcal vaccination, or risk for exposure to pneumococcal serotypes contained in PCV20 (Box).

BOX **Considerations for shared clinical decision-making regarding use of 20-valent pneumococcal conjugate vaccine in adults aged ≥65 years who have completed their recommended vaccine series with 13-valent pneumococcal conjugate vaccine and 23-valent pneumococcal polysaccharide vaccine**

- 20-valent pneumococcal conjugate vaccine (PCV20) is a safe and immunogenic vaccine for older adults. The risk for 13-valent pneumococcal conjugate vaccine (PCV13)-type disease among adults aged ≥65 years is much lower than it was before the pediatric program was implemented as a result of indirect PCV13 effects (by preventing carriage and, thereby, reducing transmission of PCV13-type strains). The effects of PCV20 use in children on adult pneumococcal disease burden are unknown.
- For these adults who have already received a dose of both PCV13 and 23-valent pneumococcal polysaccharide vaccine (PPSV23), the remaining risk depends on each patient’s risk for exposure to serotypes contained in PCV20, presence of underlying medical conditions or risk factors that increase the risk for developing pneumococcal disease or developing severe manifestation from disease, and time since last pneumococcal vaccination.
- Incidence of PCV20-type invasive pneumococcal disease and pneumonia increases with increasing age and is higher among persons who have more than one chronic medical condition,* a cerebrospinal fluid leak, a cochlear implant, or an immunocompromising condition.^†^ Although indirect effects from pediatric PCV13 use were documented for these groups of adults with underlying conditions that increase the risk for pneumococcal disease, the residual PCV13-type disease burden remains higher in these groups.^§^ There might be added benefit from PCV20 vaccination for these persons at increased risk for pneumococcal disease, especially if time has elapsed (i.e., ≥5 years) since last receipt of pneumococcal vaccine.

* Alcoholism; chronic heart, liver, or lung disease; cigarette smoking; or diabetes mellitus.

^†^ Immunocompromising conditions are defined as chronic renal failure, nephrotic syndrome, immunodeficiency, iatrogenic immunosuppression, generalized malignancy, HIV infection, Hodgkin disease, leukemia, lymphoma, multiple myeloma, solid organ transplant, congenital or acquired asplenia, or sickle cell disease or other hemoglobinopathies.

^§^ **Source:** Ahmed SS, Pondo T, Xing W, et al. Early impact of 13-valent pneumococcal conjugate vaccine use on invasive pneumococcal disease among adults with and without underlying medical conditions—United States. Clin Infect Dis 2020;70:2484–92.

#### Rationale

Phase III clinical trial data among adults aged ≥65 years without immunocompromising conditions who received both PCV13 and PPSV23 1–5 years previous indicated that PCV20 use is safe and immunogenic (https://www.cdc.gov/vaccines/acip/recs/grade/PCV20-prev-vax-older-adults-etr.html). Economic models demonstrated that cost per QALY gained of recommending PCV20 for adults aged ≥65 years who received both PCV13 and PPSV23 ranged from $93,000 per QALY gained (Pfizer model: 7 years after receiving both PCV13 and PPSV23 at age 65 years) to $970,000 per QALY gained (Tulane-CDC model: 1 year after receiving both PCV13 and PPSV23 at age 65 years). Whereas certain adults might benefit from additional protection against pneumococcal pneumonia after completing the previously recommended pneumococcal vaccine series with both PCV13 and PPSV23, shared clinical decision-making, rather than routine use, is recommended regarding PCV20 for this population on the basis of findings from the economic analyses.

## Recommendations for Use of Pneumococcal Vaccines in Adults Aged 19–64 Years With Certain Underlying Medical Conditions or Other Risk Factors

### Adults Who Previously Have Not Received Any Pneumococcal Vaccine

Adults aged 19–64 years with certain underlying medical conditions or other risk factors (i.e., alcoholism; chronic heart, liver, or lung disease; chronic renal failure; cigarette smoking; cochlear implant; congenital or acquired asplenia; CSF leak; diabetes mellitus; generalized malignancy; HIV infection; Hodgkin disease; immunodeficiency; iatrogenic immunosuppression; leukemia, lymphoma, or multiple myeloma; nephrotic syndrome; solid organ transplant; or sickle cell disease or other hemoglobinopathies) who previously have not received a pneumococcal vaccine, or whose previous vaccination history is unknown, should receive a single dose of PCV20 or PCV15. When PCV15 is used, it should be followed at a ≥1 year interval with a single dose of PPSV23 (Tables 3, 4, 5, and 7). The minimum recommended interval for receipt of the PPSV23 dose in adults with an immunocompromising condition, a CSF leak, or a cochlear implant is ≥8 weeks since the PCV15 dose.

#### Rationale

Phase II and III clinical trials indicated that the safety and immunogenicity of PCV15 used in series with PPSV23 in adults with underlying medical conditions or other risk factors for pneumococcal disease were comparable with those of PCV13 use in series with PPSV23 (*23*). PCV20 clinical trials included adults with stable chronic medical conditions but did not include adults with immunocompromising conditions (*21*). A post hoc analysis of a PCV13 RCT among adults aged ≥65 years found that PCV13 is effective in preventing VT-IPD (*157*) or VT-pneumococcal pneumonia (*158*) among adults with stable chronic medical conditions. Limited PCV13 effectiveness data from observational studies that included adults with immunocompromising conditions demonstrate that PCV13 confers protection against VT-IPD (*147*–*149*) or VT-pneumococcal pneumonia (*151*). Economic models assessing use of PCV20 alone or PCV15 in series with PPSV23 demonstrated that compared with the previous recommendations, the new interventions will prevent more disease with an associated increase in cost (*208*,*209*).

### Adults With an Immunocompromising Condition, a CSF Leak, or a Cochlear Implant Who Previously Have Received PCV13 Only

Adults aged 19–64 years with an immunocompromising condition (i.e., chronic renal failure, nephrotic syndrome, immunodeficiency, iatrogenic immunosuppression, generalized malignancy, HIV infection, Hodgkin disease, leukemia, lymphoma, multiple myeloma, solid organ transplant, congenital or acquired asplenia, or sickle cell disease or other hemoglobinopathies), a CSF leak, or a cochlear implant who previously have received PCV13 only are recommended to receive either a single dose of PCV20 ≥1 year after the PCV13 dose or a single dose of PPSV23 at a ≥8 week interval after the PCV13 dose (*11*,*14*) (Tables 3 and 4). When PPSV23 is used instead of PCV20 for these adults, either PCV20 or a second PPSV23 dose is recommended ≥5 years after the first PPSV23 dose for adults with an immunocompromising condition but not for adults with a CSF leak or a cochlear implant (Table 3). Review the pneumococcal vaccine recommendations again when the person turns age 65 years (Tables 3 and 4). If PCV20 is used in place of any dose of PPSV23, the series is complete and it need not be followed by additional pneumococcal vaccines. For adults aged 19–64 years who do not have an immunocompromising condition, a CSF leak, or a cochlear implant who previously have received PCV13 only, refer to new CDC guidelines for implementation (see New CDC Guidance).

#### Rationale

Phase III clinical study data among adults aged ≥65 years without immunocompromising conditions indicated that PCV20 use in adults who received PCV13 ≥6 months previous was safe and immunogenic (https://www.cdc.gov/vaccines/acip/recs/grade/PCV20-prev-vax-adults-etr.html). Although no data are available on PCV20 use among adults with immunocompromising conditions who previously received PCV13, repeat PCV13 doses have been demonstrated to be modestly immunogenic in adults with immunocompromising conditions (*183*,*184*). In a cohort of adults aged 42 years (age selected to represent adults aged 19–64 years) with immunocompromising conditions, the cost per QALY gained of PCV20 use 1 or 5 years after receipt of PCV13 compared with no vaccine use was $279,000 with a fifth and 95th percentile range of $197,000–$540,000 in probabilistic sensitivity analyses. Although PPSV23 provides the broadest serotype coverage among available pneumococcal vaccines, considering the immunologic advantages of PCVs compared with PPSV23 (*113*–*115*), use of a single dose of PCV20 is an option to PPSV23 using previously recommended doses and intervals for adults who have received PCV13 to provide broader pneumococcal serotype coverage.

### Adults With an Immunocompromising Condition, a CSF Leak, or a Cochlear Implant Who Previously Have Received Both PCV13 and PPSV23

Adults aged 19–64 years with an immunocompromising condition (i.e., chronic renal failure, nephrotic syndrome, immunodeficiency, iatrogenic immunosuppression, generalized malignancy, HIV infection, Hodgkin disease, leukemia, lymphoma, multiple myeloma, solid organ transplant, congenital or acquired asplenia, or sickle cell disease or other hemoglobinopathies), a CSF leak, or a cochlear implant who have received both PCV13 and PPSV23 but have not received the full recommended vaccination series are recommended to complete their pneumococcal vaccine series by receiving either a single dose of PCV20 ≥5 years after the last pneumococcal vaccine dose or ≥1 dose of PPSV23. When a second PPSV23 dose is used instead of PCV20, it should be administered ≥8 weeks after the PCV13 dose and ≥5 years after the first PPSV23 dose for adults with an immunocompromising condition but not for adults with a CSF leak or a cochlear implant (Table 3). Review the pneumococcal vaccine recommendations again when the person turns age 65 years (Tables 3 and 4). If PCV20 is used in place of any dose of PPSV23, the series is complete and it need not be followed by additional pneumococcal vaccines.

#### Rationale

Phase III clinical trial data among adults aged ≥65 years without immunocompromising conditions who received both PCV13 and PPSV23 1–5 years previous indicated that PCV20 use is safe and immunogenic (https://www.cdc.gov/vaccines/acip/recs/grade/PCV20-prev-vax-adults-19-64-risk-based.html). Economic models that assessed use of PCV20 for immunocompromised adults aged <65 years who previously have received PCV13 and PPSV23 had variable results, ranging from $40,000 per QALY gained (Pfizer model: PCV20 administered 7 years since the PCV13 dose, assuming no indirect effects from pediatric PCV20 vaccination) to higher cost and worse health outcomes (Merck model: PCV20 use at 5 years after receipt of both PCV13 and PPSV23) because of differences in the model assumptions. Although PPSV23 provides the broadest serotype coverage among available pneumococcal vaccines, considering the immunologic advantages of PCVs compared with PPSV23 (*113*–*115*), use of a single dose of PCV20 is an option to PPSV23 using previously recommended doses and intervals for adults who have received PCV13 to provide broader pneumococcal serotype coverage for these adults who are at increased risk for pneumococcal disease. Adults aged <65 years who do not have an immunocompromising condition, a CSF leak, or a cochlear implant were not recommended to receive PCV13 in series with PPSV23 (*14*).

##  CDC Guidance for Implementation

Updated and new CDC guidance for implementation was presented to the ACIP but did not go for a full vote. The intent of these guidelines was to provide guidance on how to apply the new recommendations for specific populations.

### Updated CDC Guidance

#### Adults Who Previously Have Received PPSV23 Only

Adults who have received PPSV23 only, including those who received PPSV23 only on the basis of previous pneumococcal vaccine recommendations or those who received PPSV23 before receiving a dose of PCV15 in the PCV15-PPSV23 series, should receive a single dose of either PCV20 or PCV15 ≥1 year after the last PPSV23 dose. When PCV15 or PCV20 is used in adults who previously have received PPSV23, it need not be followed by another dose of PPSV23 (Tables 2, 3, 4, and 5).

##### Rationale

In 2019, ACIP discontinued the recommendations for routine PCV13 use in adults aged ≥65 years without an immunocompromising condition, a CSF leak, or a cochlear implant because of the limited population-level impact of the recommendation (*14*). PCV15 and PCV20 contain additional serotypes that are not contained in PCV13, and considering the immunologic advantages of PCVs compared with PPSV23 (*113*–*115*) as well as the limited duration of protection from PPSV23 (*129*,*137*,*138*,*140*,*141*), either a single dose of PCV15 or PCV20 is now recommended for all adults who previously received PPSV23 only.

### New CDC Guidance

#### Dosing Schedule for PCV15 and PPSV23 Series

When PCV15 is used, the recommended interval between administration of PCV15 and PPSV23 is ≥1 year. A minimum interval of 8 weeks can be considered for adults with an immunocompromising condition, a CSF leak, or a cochlear implant to minimize the time of risk for IPD caused by serotypes unique to PPSV23 in these groups at increased risk for pneumococcal disease (*17*). PCV15 and PPSV23 should not be administered during the same visit. If a dose of PPSV23 is inadvertently administered before a dose of PCV15 is administered, it should be followed by a single dose of PCV15 or PCV20 ≥1 year after the PPSV23 dose.

#### Adults Who Have Received PCV7 Only

Adults who have received PCV7 only, either as a child or inadvertently as an adult, should be vaccinated per the ACIP recommendations for adults aged ≥19 years who previously have not received any pneumococcal vaccine. This is because PCV7 provides protection against a small proportion of current pneumococcal disease.

#### Adults Aged 19–64 Years With Chronic Medical Conditions Who Have Received PCV13 Only

Adults aged 19–64 years with chronic medical conditions (i.e., alcoholism; chronic heart disease, including congestive heart failure and cardiomyopathies; chronic liver disease; chronic lung disease, including chronic obstructive pulmonary disease, emphysema, and asthma; cigarette smoking; or diabetes mellitus) were not recommended to receive PCV13 under the previous recommendations (*14*). These adults previously were recommended to receive a dose of PPSV23 only. However, if an adult received a dose of PCV13, either a single dose of PCV20 or PPSV23 should be administered ≥1 year after the PCV13 dose to provide pneumococcal serotype coverage (Table 5). The pneumococcal vaccine recommendations should be reviewed again when the person turns age 65 years (Table 2).

#### Adults Aged 19–64 Years With Chronic Medical Conditions Who Have Received Both PCV13 and PPSV23

According to previous recommendations (*14*), adults aged 19–64 years with chronic medical conditions (i.e., alcoholism; chronic heart disease, including congestive heart failure and cardiomyopathies; chronic liver disease; chronic lung disease, including chronic obstructive pulmonary disease, emphysema, and asthma; cigarette smoking; or diabetes mellitus) were not recommended to receive PCV13 in series with PPSV23. Instead, these adults previously were recommended to receive a single dose of PPSV23 only. If such an adult received both PCV13 and PPSV23, no additional pneumococcal vaccine dose is indicated (Table 5). The pneumococcal vaccine recommendations should be reviewed again when the person turns age 65 years (Table 2).

#### Adults Who Received HSCT

Adults who received HSCT are recommended to receive 3 doses of PCV20, 4 weeks apart starting 3–6 months after HSCT. A fourth PCV20 dose is recommended ≥6 months after the third dose of PCV20 or ≥12 months after HSCT, whichever is later. HSCT recipients who have started their pneumococcal vaccine series with PCV13 or PCV15 can complete their 4-dose pneumococcal vaccine series with PCV20 without receiving extra doses. If PCV20 is not available, 3 doses of PCV15 followed by 1 dose of PPSV23 ≥12 months after HSCT can be administered. For patients with chronic GVHD who are receiving PCV15, a fourth dose of PCV15 can be administered in place of PPSV23 because these adults are less likely to respond to PPSV23. A patient’s clinical team is best informed to determine the appropriate timing of vaccination (Table 6).

##### Rationale

HCST recipients have a poor response to PPSV23 when it is administered during the first year after transplantation or later, especially those with chronic GVHD. Although no clinical trial data are available on PCV20 use among HSCT recipients, studies that assessed the use of 4 doses of PCV13 (3 doses and 1 booster dose) followed by 1 dose of PPSV23 demonstrated that use of 4 doses of PCV13 is immunogenic (*193*,*194*), and PCV20 provides the broadest serotype coverage among available PCVs. Phase III clinical trial data indicated that the immunogenicity of PCV15 was comparable with that of PCV13 when used either with a dose of PPSV23 (in those without GVHD) or PCV (in those with GVHD) as the fourth dose (*188*). The frequency of vaccine-related adverse events was higher after use of 3 doses of PCV15, although the majority of adverse events were mild to moderate.

#### Coadministration With Other Vaccines

In accordance with ACIP’s General Best Practice Guidelines for Immunization, routine administration of a pneumococcal vaccine with other age-appropriate doses of vaccines at the same visit is recommended for adults who have no specific contraindications at the time of the health care visit (*210*). For example, coadministration with a COVID-19 vaccine can be administered at a different anatomic site during the same visit. More information on coadministration of COVID-19 vaccine is detailed in the Interim Clinical Considerations for Use of COVID-19 Vaccines Currently Approved or Authorized in the United States (*211*).

## Precautions and Contraindications

Before administering a pneumococcal vaccine, vaccination providers should consult the package insert for precautions, warnings, and contraindications (*116*–*118*,*120*). Vaccination with a PCV or PPSV23 is contraindicated in persons known to have a severe allergic reaction (e.g., anaphylaxis) to any component of the vaccine. In addition, PCVs are contraindicated in persons known to have a severe allergic reaction to any diphtheria toxoid–containing vaccine. Before pneumococcal vaccine administration, all precautions should be taken to prevent allergic or any other adverse reactions. These precautions should include a review of the patient’s vaccination history for possible sensitivity to the vaccine or similar vaccines and for previous vaccination-related adverse reactions to determine the presence of any contraindication to vaccination with a pneumococcal vaccine and to allow for an assessment of risks and benefits, as described in ACIP’s General Best Practice Guidelines for Immunization (*212*).

### Pregnancy

ACIP has not made any recommendations on use of pneumococcal vaccines for pregnant persons. Pregnancy is associated with decreased CD4 T-cell counts and mild immune suppression, suggesting that vaccines might not achieve optimal immunogenicity (*213*). Most of the available information on pneumococcal vaccine use during pregnancy relates to PPSV23 (*214*–*227*).

#### Safety

Multiple RCTs among pregnant women have found that vaccine-related SAEs in women vaccinated with PPSV23 are rare (*214*,*220*–*227*), and the frequency of pregnancy-related adverse outcomes (e.g., stillbirth or low birthweight) or deaths among infants born to mothers vaccinated with PPSV23 were comparable with those observed among the control groups (*215*,*216*,*220*–*225*,*227*). In comparison, data on PCV use in pregnant women are limited. One clinical trial in Brazil randomized pregnant women (13 weeks’ to <34 weeks’ gestational age) with HIV infection on antiretroviral therapy to receive either 10-valent pneumococcal conjugate vaccine (PCV10), PPSV23, or placebo (*219*). The percentage of pregnant women who developed treatment-related grade ≥3 events (i.e., severe events) within 4 weeks of vaccination (1% in each group) and percentage who developed grade ≥4 events (i.e., potentially life-threatening events) >4 weeks postvaccination (2% in PCV10 group, 1% in PPSV23 group, and 3% in placebo group) were comparable across the three groups (*217*,*228*). The percentage of adverse pregnancy outcomes also was similar across the groups, except for lower proportion of preterm births in the PCV10 group (2%; two of 98) compared with the PPSV23 (13%; 13 of 98) and placebo (12%; 12 of 103) groups (*217*).

#### Immunogenicity

Studies have found that PPSV23 increases the serotype-specific antipneumococcal antibody concentration in the serum after vaccination of pregnant women (*214*,*215*,*221*,*225*,*227*). Antipneumococcal antibody concentrations also were higher in cord blood of women vaccinated with PPSV23 compared with women not vaccinated with PPSV23 (*214*,*215*,*221*,*225*,*227*) and in serum of infants who were born to mothers who received PPSV23 compared with those born to mothers who did not receive PPSV23 (*215*,*218*,*225*,*227*). However, the infants’ increased antibody concentrations decreased during the first few months of life.

In the RCT of pregnant women with HIV infection in Brazil, seroresponses (defined as greater than twofold increase in antibodies from baseline to 28 days after vaccination) against five or more serotypes were similar between women who received PCV10 (65%) and PPSV23 (65%) (*217*). At birth, the percentage of infants who had seroprotection (defined as antipneumococcal antibody concentration of ≥0.35 *μ*g/mL) against five or more serotypes was 67% in infants born to mothers who received PCV10, 57% in infants born to mothers who received PPSV23, and 17% in infants in the placebo group. At age 8 weeks, the percentage with seroprotection decreased to 19%, 23%, and 1% in PCV10, PPSV23, and placebo groups, respectively (*217*). When immunogenicity of PCV10 and PPSV23 administered antepartum and postpartum were compared, results indicated that antibody concentrations were higher among women who were vaccinated postpartum (*213*). The trial further assessed the immunogenicity to PCV10 in infants born to mothers who received either PCV10 or PPSV23 during pregnancy (*219*). When immunogenicity was assessed 1–3 months after the second infant dose of PCV10, infants born to mothers who received PCV10 had significantly lower antibody levels against five of seven common serotypes compared with infants born to mothers who received PPSV23, and the seroprotection rate against seven common serotypes was statistically significantly different with 50% seroprotection rate in the maternal PCV10 group compared with 71% in the maternal PPSV23 group.

### Reporting of Vaccine Adverse Events

Before administering PCV15, PCV20, or PPSV23, health care providers should consult relevant package inserts (*116*,*117*,*120*) regarding precautions and contraindications. Adverse events occurring after administration of any vaccine should be reported to the Vaccine Adverse Event Reporting System (VAERS). Additional information about VAERS and how to report an adverse event is available at https://vaers.hhs.gov.

### National Vaccine Injury Compensation Program

The National Vaccine Injury Compensation Program (VICP), established by the National Childhood Vaccine Injury Act of 1986, as amended, is a mechanism through which compensation can be provided to persons who might have been injured as a result of receiving a vaccine covered by VICP. The Vaccine Injury Table (https://www.hrsa.gov/sites/default/files/hrsa/vicp/vaccine-injury-table-01-03-2022.pdf) lists the vaccines covered by VICP and the associated injuries and conditions that might receive a legal presumption of causation. If the injury or condition is not in the table or does not meet the requirements in the table, persons must prove that the vaccine caused the injury or condition. Claims must be filed with specified time frames. Adults of all ages who receive a VICP-covered vaccine might be eligible to file a claim. Additional information is available at https://www.hrsa.gov/vaccine-compensation or by calling 1-800-338-2382.

## Future Directions

CDC and ACIP will continue to assess safety of PCV15 and PCV20 use in adults, monitor the impact of implementing these new recommendations, and assess postimplementation pneumococcal disease trends. These vaccines were licensed on the basis of safety and immunogenicity data, and correlates of protection have not been established for adults.

Multiple factors might influence future pneumococcal vaccination strategies for adults. Unlike PCV13, the new PCVs (PCV15 and PCV20) were first licensed by FDA for use in adults. PCV15 use in children was approved by FDA and recommended by ACIP in June 2022 (*229*), and PCV20 use was approved by FDA in April 2023 and recommended by ACIP in June 2023 (*230*). Similar to observations with PCV13, indirect effects from use of these new PCVs in children might reduce the added benefit of PCV15 or PCV20 vaccination in adults in the future (*14*). In addition, new, higher-valency pneumococcal vaccines for adults have completed phase II clinical trials, and these vaccines might become available for use in upcoming years (*231*–*233*). Furthermore, the long-term impact of COVID-19 on pneumococcal disease burden in the United States is unknown.

Adult pneumococcal vaccine recommendations have become more complicated as more pneumococcal vaccines have become available for use. Pneumococcal vaccine coverage, especially among adults aged <65 years with underlying conditions that increase the risk for pneumococcal disease, remains suboptimal (*50*,*207*,*234*). Future availability of vaccines that cover broader serotypes might provide an opportunity to simplify the adult pneumococcal vaccine recommendations and increase vaccine coverage or expand the risk-based recommendations to maximize protection against pneumococcal disease. ACIP will review and update recommendations as new data become available.

## Footnotes

* Serotypes 15B and 15C are identified together as 15B/15C in the SSUAD test used in this study.

† Primary noninferiority hypothesis was lower bound of the two-sided 95% CI of the IgG GMC ratio (PCV15/PPSV23) of the 14 shared serotypes to be >0.5; secondary noninferiority hypothesis was lower bound of the two-sided 95% CI of the OPA GMT ratio (PCV15/PPSV23) of the 14 shared serotypes to be >0.33.

§ Lower bound of the two-sided 95% CI of the OPA GMT ratio (PCV15/PCV13) to be >0.5.

¶ For PCV15-unique serotypes 22F and 33F, lower bound of the two-sided 95% CI of the OPA GMT ratio (PCV15/PCV13) to be >2.0 and the lower bound of the two-sided 95% CI of the differences (PCV15 − PCV13) between the percentages of participants with at least a fourfold rise to be >0.1. For serotype 3, lower bound of the two-sided 95% CI of the OPA GMT ratio (PCV15/PCV13) to be >1.2 and the lower bound of the two-sided 95% CI of the differences (PCV15 − PCV13) between the percentages of participants with at least a fourfold rise to be >0.

** Range reflects the difference in results across studies. For example, in one study, the group that received PCV15 may have numerically similar or higher OPA geometric mean antibody titers for nine of 13 shared serotypes compared with the group that received PCV13. In another study, the group that received PCV15 might have numerically similar or higher OPA GMT for 13 of 13 shared serotypes. The specific serotypes with numerically similar or higher OPA GMT might vary.

†† These incremental cost-effectiveness ratios (PCV15 + PPSV23 versus early 2021 recommendations) were not directly reported in the study, but were calculated on the basis of costs and QALYs reported in Smith KJ, Wateska AR, Nowalk MP, et al. Higher-valency pneumococcal conjugate vaccines: an exploratory cost-effectiveness analysis in U.S. seniors [Table 2]. Am J Prev Med 2021;61:28–36.
